# Multi-level analysis of gut microbiome extracellular vesicles-host interaction reveals a connection to gut-brain axis signaling

**DOI:** 10.1128/spectrum.01368-24

**Published:** 2024-12-19

**Authors:** Walid Mottawea, Basit Yousuf, Salma Sultan, Tamer Ahmed, JuDong Yeo, Nico Hüttmann, Yingxi Li, Nour Elhouda Bouhlel, Hebatoallah Hassan, Xu Zhang, Zoran Minic, Riadh Hammami

**Affiliations:** 1 NuGut Research Platform, School of Nutrition Sciences, Faculty of Health Sciences, University of Ottawa, Ottawa, Canada; 2 Department of Microbiology and Immunology, Faculty of Pharmacy, Mansoura University, Mansoura, Egypt; 3 John L. Holmes Mass Spectrometry Facility, Faculty of Science, University of Ottawa, Ottawa, Canada; 4 Regulatory Research Division, Centre for Oncology, Radiopharmaceuticals and Research, Biologic and Radiopharmaceutical Drugs Directorate, Health Products and Food Branch, Health Canada, Ottawa, Canada; 5 Department of Biochemistry, Microbiology and Immunology, Faculty of Medicine, University of Ottawa, Ottawa, Canada; University of Nebraska-Lincoln, Lincoln, Nebraska, USA

**Keywords:** gut microbiome, extracellular vesicles, neuroactive metabolites, gut-brain axis, *Bacteroides*, γ-aminobutyric acid (GABA)

## Abstract

Microbiota-released extracellular vesicles (MEVs) have emerged as a key player in intercellular signaling. However, their involvement in the gut-brain axis has been poorly investigated. We hypothesize that MEVs cross host cellular barriers and deliver their cargoes of bioactive compounds to the brain. In this study, we aimed to investigate the cargo capacity of MEVs for bioactive metabolites and their interactions with the host cellular barriers. First, we conducted a multi-omics profiling of MEVs’ contents from *ex vivo* and stool samples. Metabolomics analysis identified various neuro-related compounds encapsulated within MEVs, such as arachidonyl-dopamine, gabapentin, glutamate, and N-acylethanolamines. Metaproteomics unveiled an enrichment of enzymes involved in neuronal metabolism, primarily in the glutamine/glutamate/gamma-aminobutyric acid (GABA) pathway. These neuro-related proteins and metabolites were correlated with *Bacteroides* spp. We isolated 18 *Bacteroides* strains and assessed their GABA production capacity in extracellular vesicles (EVs) and culture supernatant. A GABA-producing *Bacteroides finegoldii*, released EVs with a high GABA content (4 µM) compared to *Phocaeicola massiliensis*. Upon testing the capacity of MEVs to cross host barriers, MEVs exhibited a dose-dependent paracellular transport and were endocytosed by Caco-2 and hCMEC/D3 cells. Exposure of Caco-2 cells to MEVs did not alter expression of genes related to intestinal barrier integrity, while affected immune pathways and cell apoptosis process as revealed by RNA-seq analyses. *In vivo*, MEVs biodistributed across mice organs, including the brain, liver, stomach, and spleen. Our results highlight the ability of MEVs to cross the intestinal and blood-brain barriers to deliver their cargoes to distant organs, with potential implication for the gut-brain axis.

**IMPORTANCE:**

Microbiota-released extracellular vesicles (MEVs) have emerged as a key player in intercellular signaling. In this study, a multi-level analysis revealed presence of a diverse array of biologically active molecules encapsulated within MEVs, including neuroactive metabolites, such as arachidonyl-dopamine, gabapentin, glutamate, and N-acylethanolamines, and gamma-aminobutyric acid (GABA). Metaproteomics also unveiled an enrichment of neural-related proteins, mainly the glutamine/glutamate/GABA pathway. MEVs were able to cross epithelial and blood-brain barriers *in vitro*. RNA-seq analyses showed that MEVs stimulate several immune pathways while suppressing cell apoptosis process. Furthermore, MEVs were able to traverse the intestinal barriers and reach distal organs, including the brain, thereby potentially influencing brain functionality and contributing to mental and behavior.

## INTRODUCTION

Appreciable evidence suggests the connection between the microbiota-gut-brain axis and the host’s brain activity (1, 2). For instance, germ-free mice exhibited a hyperactive hypothalamus-pituitary axis and higher levels of stress hormones than conventional mice (3). Also, the gut microbiota has been shown to regulate emotional behaviors and modulate anxiety and memory processing (4–6). Some probiotics, known as “psychobiotics,” demonstrate psychotropic-like activities and are suggested to modulate mental and behavioral disorders. While some studies have reported mitigated efficacy, several trials support the role of psychobiotics in the normalization of brain processes related to stress responses and mood improvements (7–12). However, the mechanisms by which psychobiotics and gut microbiota interact with the gut-brain axis and modulate mental health remain hypothetical.

Microbiota-released extracellular vesicles (MEVs) have recently emerged as signaling molecules that mediate host-microbiota crosstalk (13). MEVs are small membrane-bound phospholipid vesicles that range from 30 nm to 1 µm in size, with larger vesicles originating from the cell surface (microvesicles/ectosomes), and those on the smaller side being derived from either the plasma membrane or the endosomal system (exosomes) (14). MEVs encase a spectrum of biologically active proteins, mRNA, miRNA, DNA, carbohydrates, lipids, and metabolites, thus propagating the horizontal transfer of their cargo across both short and long distances (13). MEVs are involved in processes such as quorum sensing, biofilm formation, relief of environmental stresses, and host immunomodulation (15). The production and role of MEVs released by probiotic and commensal microbes in the gut environment are poorly investigated (16), as MEVs have been predominantly examined in individual strains (15, 17, 18). Previously, Kang et al. (19) reported an important shift in stool MEVs composition compared to the microbiome in a dextran sulfate sodium (DSS)-induced colitis mouse model. Moreover, the same authors observed an attenuating effect of *Akkermansia muciniphila*-derived MEVs on colitis severity (19), suggesting the potential of MEVs as a biomarker and therapeutic agent. The recent report on increased levels of systemic lipopolysaccharide (LPS)-positive bacterial extracellular vesicles (EVs) in patients with intestinal barrier dysfunction provides some evidence on the capacity of MEVs to circulate throughout a system (20), and elicit a variety of immunological and metabolic responses in different organs, including the brain. Therefore, MEVs should be considered important delivery vehicles for host-modulating metabolites, and thus could be regarded as promising psychobiotic candidates compared to the clinical and regulatory limitations faced by other approaches such as fecal transplantation (21, 22).

Harnessing the MEVs production ability of gut microbiota could decipher interactions with the gut-brain axis. In the current study, we postulate that microbiota interplays with the gut-brain axis involves MEVs as a potential cargo mechanism by which gut microbiota deliver their bioactive metabolites to the brain. Given that fecal EVs originate from both microbiome cells and host cells, we investigated MEVs released by *ex vivo*-developed microbiomes, along with MEVs obtained from stool samples of healthy adults, to segregate the potential confounding effects of EVs released by host cells.

## RESULTS

### Stool and *ex vivo*-developed gut microbiota structure

We characterized the stool and *ex vivo*-developed microbiota structures using 16S rRNA gene sequencing. The stool samples showed higher diversity compared to the *ex vivo*-developed microbiome (Fig. S1A, *P* < 0.0001). The effect of the donor on the microbiome structure was evident as principal coordinates analysis (PCoA) analyses showed sample clustering predominantly by sample type (stool vs *ex vivo*, Permutational multivariate analysis of variance (PERMANOVA) *P* < 0.001) and then by donor (Fig. S1B). The microbiota-developed *ex vivo* followed the common structure of the human gut microbiome, which is dominated by Bacteroidetes and Firmicutes, with a lower abundance of Proteobacteria and Actinobacteria and low percentages for other phyla (Fig. S1C). The *ex vivo*-developed microbiome showed depletion of *Ruminococcaceae* and *Lachnospiraceae* along with an expansion of *Enterobacteriaceae* and *Veillonellaceae* (Fig. S1D) compared to the stool samples. A total of 110 taxonomic features showed differential abundances between stool and *ex vivo* microbiota (Fig. S1E; Table S1). These features are dominated by *Faecalibacterium* spp. (Amplicon Sequence Variant ASV1), *Ruminococcaceae* family (ASV2) where *Gemmiger formicilis* (ASV3) is most commonly found, and *Lachnospiraceae* (ASV4) where the *Clostridium* genus (ASV5) is most prevalent.

### Extracellular vesicles generated by stool and *ex vivo*-developed microbiota (MEVs) are packed with a wide array of metabolites, including neuroactive compounds

**Fig 1:**
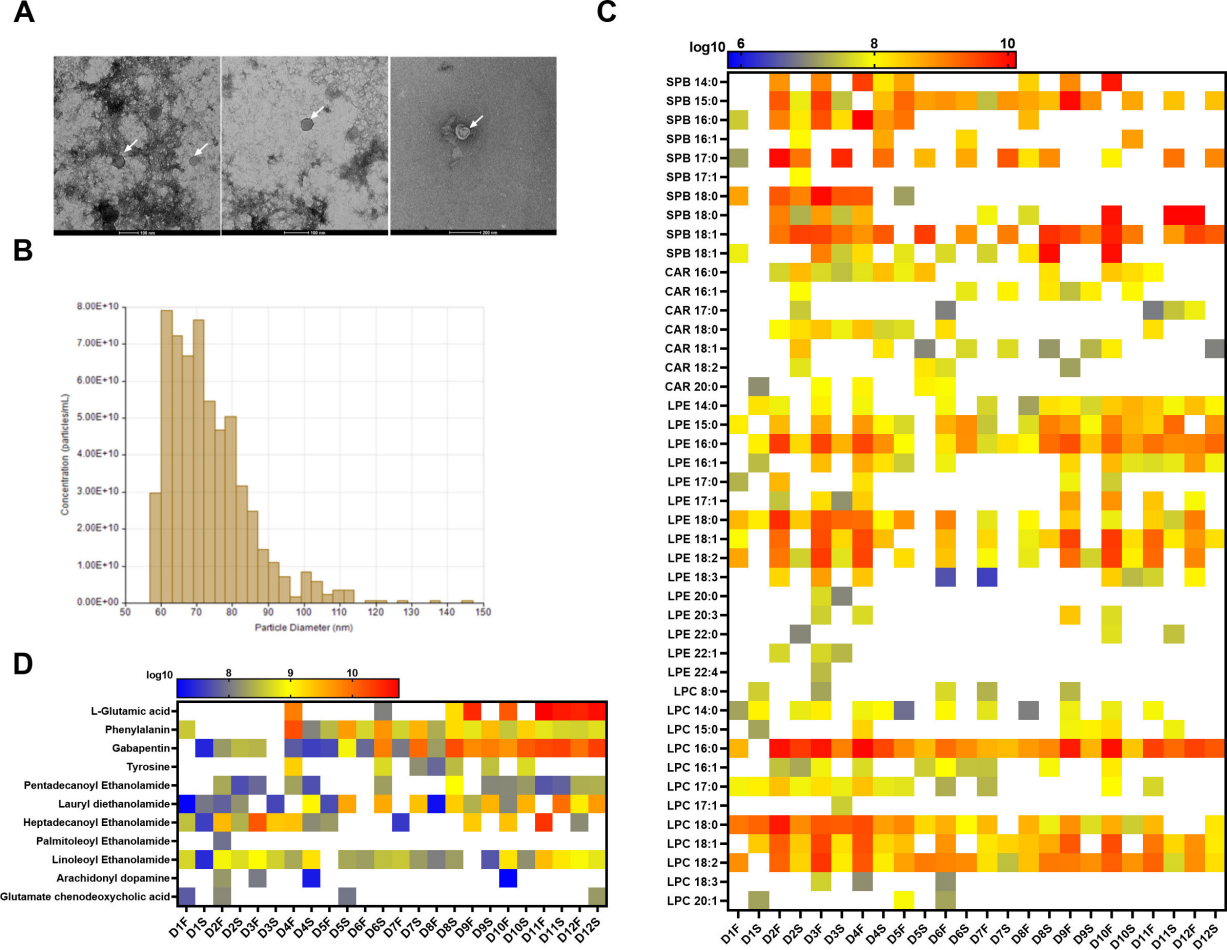
Physical and metabolic characteristics of MEVs. (**A**) TEM image depicting isolated vesicles. TEM pictures presented are for MEVs isolated from D2S, D2F, and D3S, respectively. (**B**) Nanoparticle size analysis of the isolated MEVs using tunable resistive pulse sensing (TRPS, IZON) showing the concentration as a function of the size range, the graph presents the particle size range of MEVs of D3F. (**C**) Lipid profiles of MEVs determined by nLC-ESI-MS/MS analysis in the positive mode. (**D** ) Identification of neuro-related metabolites within MEVs using nLC-ESI-MS/MS analysis in the positive mode.

Transmission electron microscopy (TEM) and Zetasizer confirmed that the isolated MEVs were intact, the membrane was enclosed, and their size fell within the reported range of exosomes, microvesicles, and bacterial outer membrane vesicles (Fig. 1A and B) (23). The MEVs from fermented samples showed more homogenous size distribution and lower size range as compared to the MEVs from stool samples (Fig. S2). First, we identified the metabolite content of MEVs by nano liquid chromatography coupled online with nanoelectrospray ionization and mass spectrometry (nLC-ESI-MS/MS) analysis in both negative and positive modes. A wide spectrum of molecules has been identified, including lipids, carbohydrates, amino acids, and steroid derivatives (Fig. S1 to S4; Tables S2 to S5).

### MEVs lipidome

First, we identified the lipid content of MEVs by nLC-ESI-MS/MS analysis in positive mode, as summarized in Fig. 1C. Over 40 lipid species were identified in the MEVs isolated from the stool and *ex vivo*-developed samples. The major lipid classes found in the samples were sphingoid bases (SPB), acylcarnitines (CAR), lyso-phosphatidylethanolamine (LPE), and lyso-phosphatidylcholine (LPC), possessing only one acyl chain in their chemical structures. A significant subset of lipid species found in MEVs contained mostly single acyl chain lipids, such as SPB, CAR, LPE, and LPC, which could serve as the primary components of the lipid bilayer membranes within MEVs (Fig. 1C; Table S2). A few phospholipid species with two acyl chains, such as phosphatidylcholine and phosphatidylethanolamine (PE), were also detected in the positive mode (not shown). Overall, MEVs isolated from fermented samples showed an increased ion peak intensity related to lipids compared to that of the stool samples.

The negative mode of nLC-ESI-MS/MS identified a total of 54 lipid species in the isolated MEVs (Fig. S3; Table S3). The dominant lipid classes found in the MEVs were LPE, and several species of N-acetylglycine, N-acyl glycylserine, PE, and phosphatidylinositol were also identified in the negative mode. Overall, different classes of lipid species were found in the negative mode compared to the positive mode, which might be due to the discrepancy in the ionization properties of each lipid class. Several lipid species that are conjugated with highly unsaturated fatty acids (HUFAs), such as linoleic acid (18:3) and arachidonic acid (20:4), were identified in the negative mode (Table S3).

### MEVs neuroactive metabolome

Other than lipids, a wide spectrum of molecules has been identified, including carbohydrates, amino acids, and steroid derivatives (Fig. S4 and S5; Tables S4 and S5). Among the metabolic components of MEVs, a set of neuroactive molecules, including choline, gabapentin, phenylalanine, tyrosine, arachidonyl-dopamine (NADA), L-glutamic acid (GA), and N-acylethanolamines, were identified in the positive mode (Fig. 1D; Table S4). In the negative mode, some neurotransmitter-related compounds were also identified. For instance, the molecular ion [M-H]^-^ of 3,4-dihydroxymandelic acid (DOMA), a metabolite of norepinephrine, referred to as a representative neurotransmitter, was identified at m/z 183.0. Additionally, arachidonyl-dopamine [M-H]^-^ was identified at m/z 438.2 and was also found in the positive mode(Tables S4 and S5). Dopamine, a prominent neurotransmitter in humans, was found in the gut MEVs in a conjugated form by association with arachidonic acid (20:4) (Fig. S5; Table S5).

**Fig 2:**
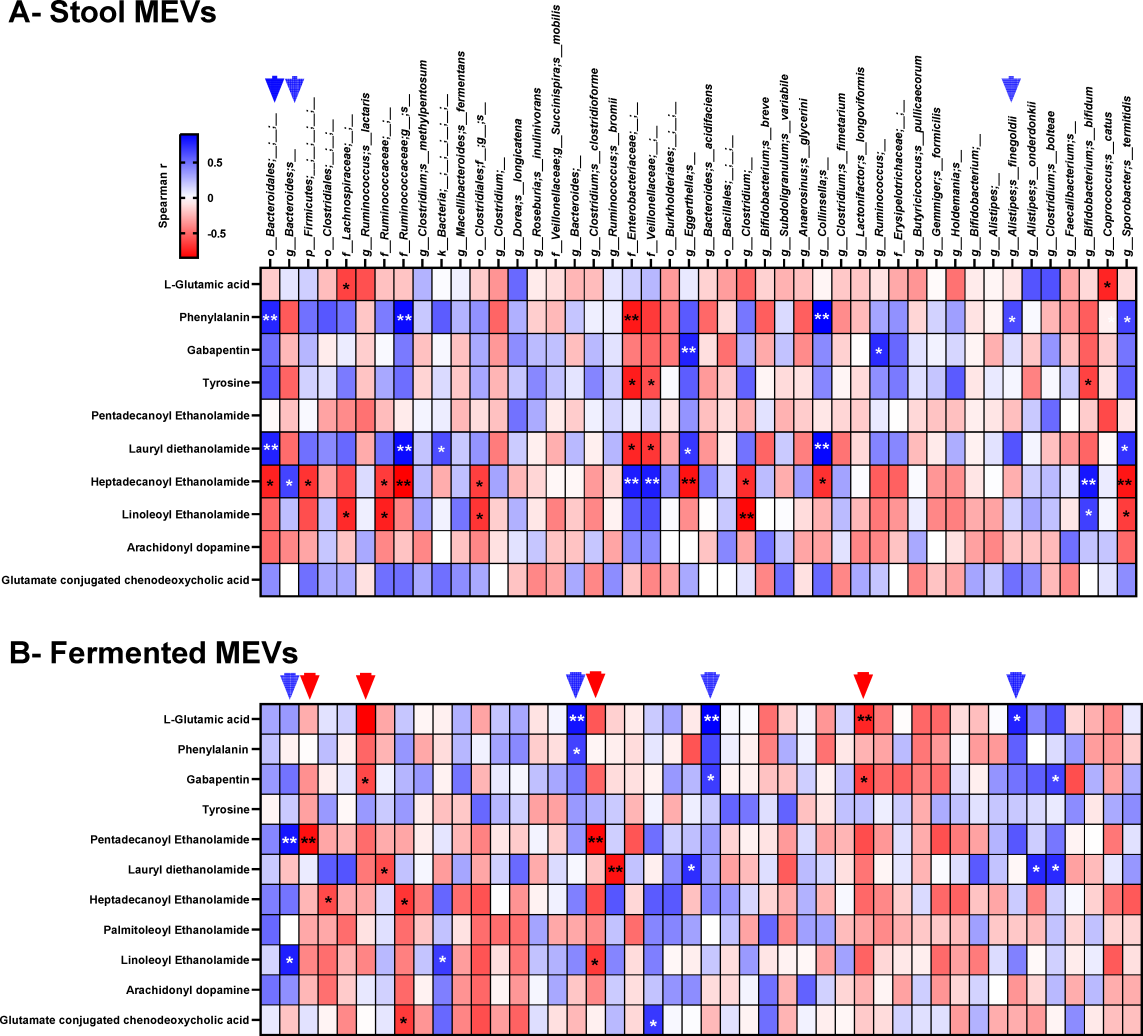
Correlation between predominant microbiota bacterial species and neuroactive metabolites. The relative abundances of microbiota bacterial species, detected in >50% of the tested samples, were correlated with the identified neuroactive metabolites from the same samples. (**A**) Correlation analysis of stool-derived MEVs. (**B**) Correlation analysis of fermented MEVs. The color scale represents the spearman correlation coefficient (*R*), and asterisks indicate the significance levels (*P < 0.05, **P < 0.01); blue arrows highlight taxa with positive correlations and red arrows highlight taxa with negative correlations.

We tested the correlation between the predominant gut bacterial species and the identified several neuroactive metabolites. We identified positive correlations between *Bacteroides* spp. and glutamic acid, phenylalanine, gabapentin, pentadecenoyl ethanolamide, and linoleoyl ethanolamide. In addition, *Alistipes* spp. showed a positive correlation with glutamic acid, gabapentin, and lauryl diethanolamide levels (Fig. 2). Conversely, *Ruminococcus* and *Clostridium* spp. were negatively correlated with identified metabolites (Fig. 2).

### MEVs are rich in proteins related to glutamine/glutamate/gamma-aminobutyric acid (GABA) pathway

**Fig 3:**
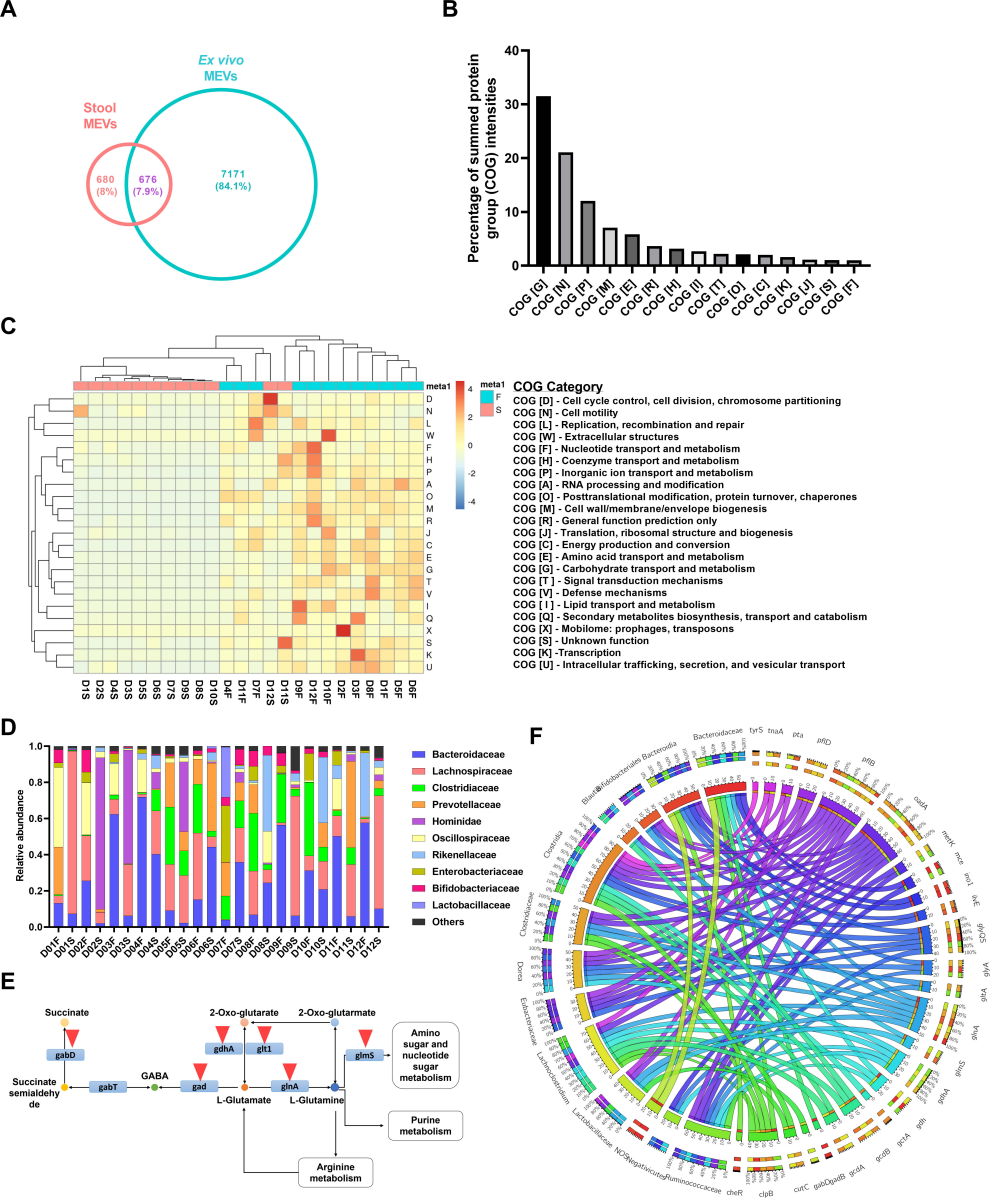
Metaproteome of MEVs is enriched in proteins with neuroactive potential. (**A**) Number of proteins identified in both stool and *ex vivo*-generated MEVs. (**B and C**) Predominance of the identified proteins at the level of COG categories (**B**) and their distribution in each sample (**C**). (**D**) Relative abundance of bacterial families identified as the source of MEVs proteome using MetaLab (25). (**E**) Glutamine/glutamate/GABA pathway is enriched in MEVs; red arrows highlight the proteins identified by the current study. (**F**) Dominant proteins with neuroactive potential and their corresponding microbiota taxa source.

Using nano-LC-MS/MS, we characterized the protein content of MEVs isolated from donor stool samples and their bioreactor-developed microbiomes. In total, 9,400 protein groups were identified, including 197 without quantitative information (Table S6). Bioreactor-generated MEVs exhibited a higher number of protein groups (7,847) than stool MEVs (1,356), with 676 protein groups shared between both groups (Fig. 3A). The bacterial exosomal marker, OmpA (24), was detected in all tested samples (Table S6). The identified proteins were classified into 15 Clusters of Orthologous Groups of proteins (COG) categories, with categories G (carbohydrate transport and metabolism), N (cell motility), P (inorganic ion transport and metabolism), and M (cell wall/membranes/envelope biogenesis) being the most abundant (Fig. 3B and C). The source taxon of the detected proteins was generated using iMetaLab (25). The major bacterial families contributing to the protein content of MEVs were *Bacteroidaceae*, *Lachnospiraceae*, *Clostridiaceae*, and *Prevotellaceae* and, to a lesser extent, other families related to Actinobacteria and Proteobacteria (Fig. 3D). Notably, we identified proteins associated with various neuroactive signaling and metabolic pathways, including the glutamine/glutamate/GABA pathway (Fig. 3E), inositol biosynthesis, microbiota-derived trimethylamine, and cellular energy production (Fig. S6; Table S7). In our data set, the proteins involved in the glutamine/glutamate/GABA pathway, such as *gadB*, *gdh*, and *glnA,* were predominantly generated by members of the *Bacteroidaceae* family, mainly *Bacteroides* (Fig. 3E and F; Table S8). Additional proteins involved in this pathway, such as *gltA* and *glmS*, were also detected in our data set and attributed to other taxa, such as *Clostridiaceae*, *Blautia,* and *Lactobacillaceae* (Fig. 3E and F; Table S8).

### Gut *Bacteroides* isolates exhibit variable GABA productivity and release EVs with distinct neurometabolic and neuroprotein content

Given the predominant association of neuroactive metabolites and proteins identified by this study with *Bacteroides* spp., we aimed to isolate different strains of *Bacteroides* that exhibit varied metabolic activities. We isolated a collection of bacterial strains derived from a fecal sample obtained from a healthy Canadian adult female. We selected 18 strains related to Bacteroidetes for further assessments of this study. For details on isolation, identification, and strain selection, please refer to the supplementary materials.

**Fig 4:**
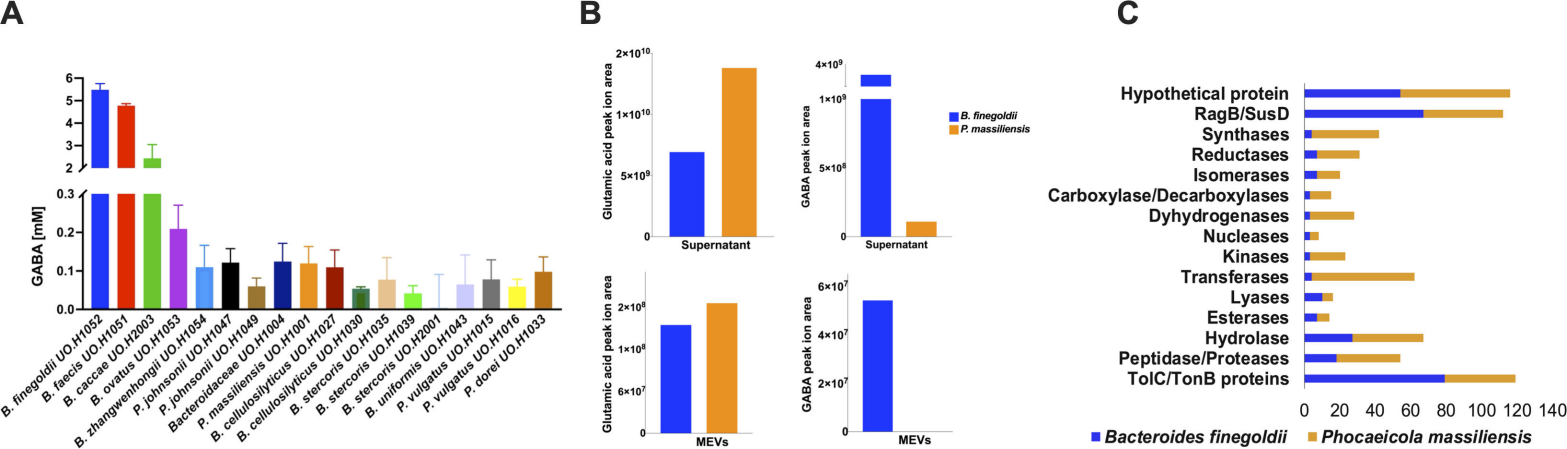
EVs released by *Bacteroides* spp. exhibit distinct neurometabolic and protein contents. (**A**) Quantification of GABA production in 18 representative *Bacteroides* spp. isolates by competitive ELISA. (**B**) Measurement of GABA and glutamic acid intensity in cell-free supernatants and EVs of *B. finegoldii* (high GABA producer) and *P. massiliensis* (low GABA producer) by nLC-nESI-MS/MS metabolomics analysis. (**C**) Identification of different enzyme categories in the proteomes of *B. finegoldii* and *P. massiliensis* using nano-LC-MS/MS proteomics analysis.

We employed a competitive enzyme-linked immunosorbent assay (ELISA) approach to assess the GABA-producing capacity among these strains. *Bacteroides finegoldii, Bacteroides faecis,* and *Bacteroides caccae* exhibited the highest GABA production in the range of 4–7 mM, followed by *Bacteroides ovatus* and *Bacteroidaceae* bacterium UO.H1004 species, in the range of 100 µM–300 µM (Fig. 4A). Other strains showed either low micromolar concentrations of GABA or no production.

EVs were isolated from two strains, the high GABA producer *B. finegoldii* UO.H1052 and the low GABA producer *Phocaeicola massiliensis* UO.H1001, to compare GABA concentrations in their supernatants and EVs. *B. finegoldii* EVs contained 4 µM GABA, whereas no GABA was detected in *P. massiliensis* EVs (Fig. S7). To further investigate the metabolic and proteomic profiles, we conducted MS/MS metabolomics and proteomics analyses of EVs and culture cell-free supernatants of *B. finegoldii* and *P. massiliensis* (Tables S9 and S10). Interestingly, glutamic acid (GA), the precursor of GABA, was identified in both EVs and the supernatant of both strains (Fig. 4B; Table S10). However, GABA was exclusively detected among the metabolites of *B. finegoldii* EVs and the supernatant, whereas it was only detected in the supernatant of *P. massiliensis*. Intriguingly, our findings revealed an inverse relationship between GABA and glutamic acid levels, underscoring the derivation of GABA from glutamic acid (Fig. 4B). We also validated the presence of GA, GABA, and tyramine in the supernatant and EVs of *B. finegoldii* by comparing fragmentation pattern with commercially available standard (Fig. S8 and S9). Additionally, our proteomic analysis of EVs and the supernatant revealed a diverse array of protein classes (Fig. 4C). Glutamate decarboxylase (GAD) was found in the EVs of both *B. finegoldii* and *P. massiliensis* and in supernatant of *P. massiliensis* only and was not detected in the supernatant of *B. finegoldii*. Glutaminase was detected in the supernatant of both *B. finegoldii*, and *P. massiliensis* and only in the EVs of *B. finegoldii* (Table S11).

### Transport of MEVs across different cell lines

**Fig 5:**
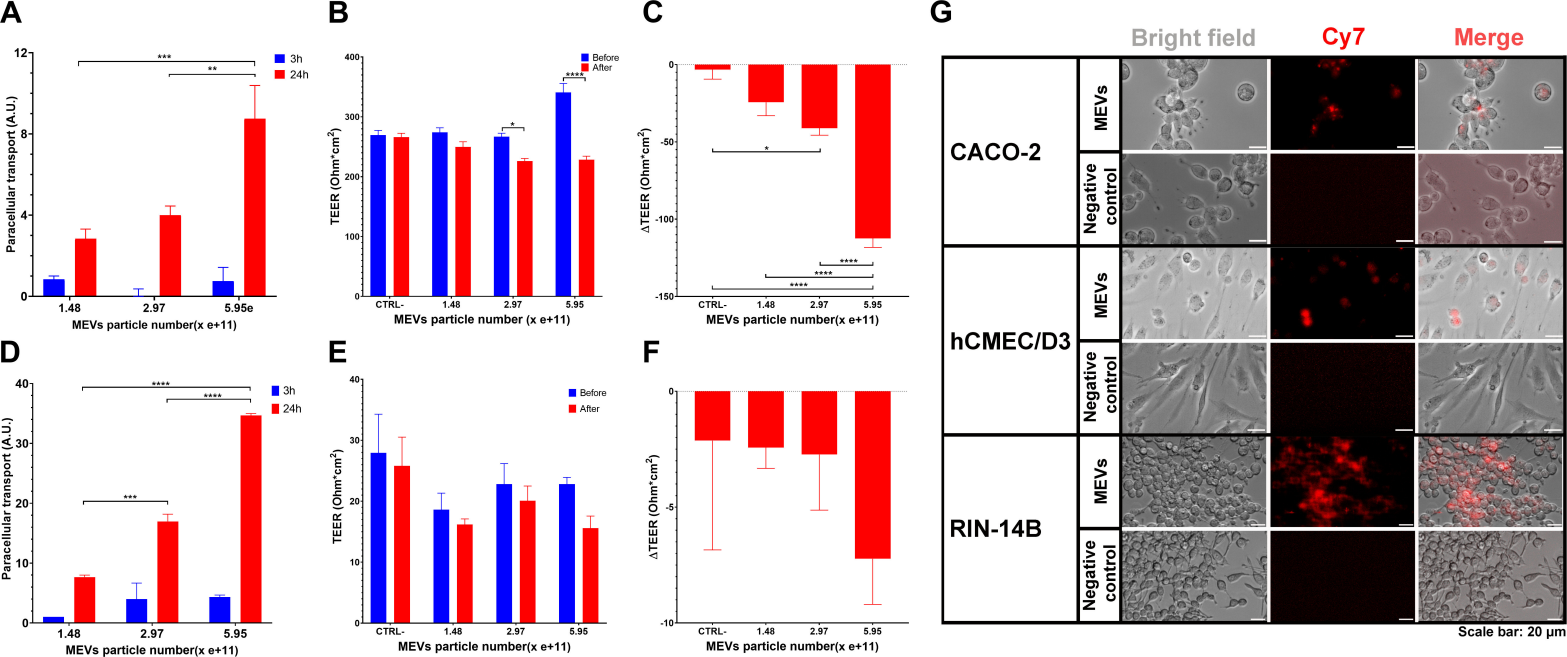
Transport of MEVs across the intestinal and blood-brain barriers via paracellular transport and endocytosis. (**A–F**) Paracellular transport of FITC-labeled MEVs at various concentrations across Caco-2 (**A**) and hCMEC/D3 (**D**) cells, presented as A.U. TEER values of Caco-2 cells (**B and C**) and hCMEC/D3 (**E and F**) before and after MEV addition. Asterisks indicate statistical significance (*P < 0.05, **P < 0.0, ***P < 0.001). (**G**) Endocytosis of Cyanine 7 (Cy7)-labeled MEVs by different cell types, including Caco-2, hCMEC/D3, and RIN-14B.

To assess the paracellular transport of MEVs across host barriers, we employed Caco-2 and hCMEC/D3 cell lines as models of intestinal and blood-brain barriers (BBBs), respectively. Fluorescein isothiocyanate (FITC)-labeled MEVs demonstrated a dose-dependent paracellular transport (expressed as arbitrary units, A.U.) across the intestinal transport model system Caco-2 cells. The transport of FITC-labeled MEVs added at a high concentration (5.95e + 11 particles, 9 ± 1.6 A.U.) was significantly higher after 24 h compared to MEVs added to the cells at low concentrations (2.97e + 11 particles, 4 ± 0.4 A.U., *P* < 0.01; 1.48e + 11 particles, 2.8 ± 0.5 A.U., *P* < 0.05) (Fig. 5A; Fig. S10). Exposure of Caco-2 cells to different concentrations of FITC-labeled MEVs for 24 h significantly decreased transepithelial electrical resistance (TEER) values (Fig. 5B and C; Table S12).

Cyanine 7 (Cy7)-labeled MEVs showed a dose-dependent paracellular transport activity across the blood-brain-barrier model system hCMEC/D3 cells. The transport of Cy7-labeled MEVs (5.95e + 11 particles, 34.7 ± 0.3 A.U.) was significantly higher after 24 h compared to labeled MEVs (2.97e + 11 particles, 17.0 ± 1.2 A.U., *P* < 0.001) and labeled MEVs (1.48e + 11 particles, 7.7 ± 0.3 A.U., *P* < 0.001). In addition, the transport of Cy7-labeled MEVs (2.97e + 11 particles) was significantly higher after 24 h compared to labeled MEVs (1.48e + 11 particles, *P* < 0.001) (Fig. 5D). Exposure of hCMEC/D3 cells to different concentrations of Cy7-labeled MEVs for 24 h did not alter the TEER values (Fig. 5E and F).

Additionally, the endocytic activity of cell lines representing models of intestinal transport (Caco-2), secretory (RIN-14B), and blood-brain barrier function (hCMEC/D3) was evaluated by adding Cy7-labeled MEVs to the respective monolayer cultures. Our results revealed that Caco-2, RIN-14B, and hCMEC/D3 cells internalized labeled MEVs via an endocytic mechanism (Fig. 5G).

### Insights from RNA-seq of Caco-2 cells

**Fig 6:**
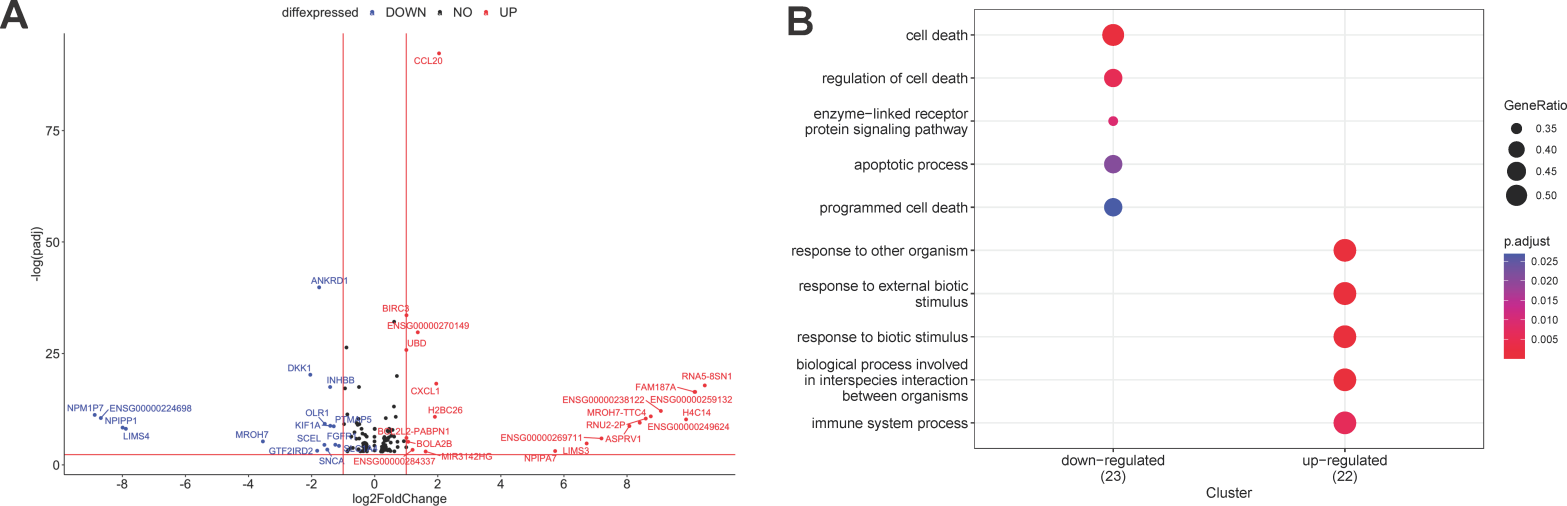
RNA-seq reveals that interaction of MEVs with Caco-2 cells modulates host immune system. (**A**) Volcano plot illustrating differentially expressed genes in MEVs-treated Caco-2 cells compared to control. The x-axis represents the log2 fold change, while the y-axis represents −log10 (*P*-value). Significance threshold is set at adjusted *P*-value ≤0.05. (**B**) Dot plot presenting enrichment results of significantly enriched biological processes and Kyoto Encyclopedia of Genes and Genomes (KEGG) pathways for upregulated and downregulated genes, with a *P*_adj_ threshold cut-off of 0.05. Enrichment significance is indicated by bubble color, while bubble size corresponds to gene count in the term.

To identify gut functionalities affected by exposure to MEVs, we conducted RNA-seq analyses of RNA from Caco-2 cells with and without MEVs exposure. The transcriptomics of Caco-2 cells following exposure to MEVs revealed a modulation in host cell functionalities. The overall RNA-seq data exhibited excellent quality, with approximately 75 million read pairs, of which 90% were mapped reads from all samples. A total of 147 genes exhibited significant (*P*_adj_ ≤ 0.05) expression differences between the MEVs-treated and control cells (Table S13). The two groups were also well-distinguished on the principal component analysis (PCA) plot. Among these genes, 22 showed significant upregulation, while 16 genes were downregulated (log fold change ≥1 or ≤ −1, *P*_adj_ ≤ 0.05) (Fig. 6A) in MEVs-treated Caco-2 cells compared to the control group.

The MEVs-treated Caco-2 cells displayed a strong enrichment of terms associated with the upregulated genes, including response to external stimuli, interspecies interaction, and immune system responses (Fig. 6B). In contrast, the downregulated genes were predominantly associated with terms related to the apoptosis process and cell death (Fig. 6B). We did not detect differential expression in terms associated with intestinal barrier integrity, implying that MEV transport does not alter the epithelial barrier functionality. Detailed enrichment analysis revealed enrichment in tumor necrosis factor (TNF), nuclear factor-kappa B (NF-κB), interleukin 17 (IL-17), IkappaB kinase signaling pathways, response to cytokines, innate immune responses, and inflammatory responses in MEVs-treated cells (Fig. S11). We also observed an upregulation of chemokines (CXCL1, CCL20) and genes associated with NF-κB signaling (BIRC3, NFKBIA, TNFAIP3, NFKB2, RELB) (Table S13). Furthermore, a substantial upregulation was noted in several genes with unknown functions.

### *In vivo* assessment of MEVs biodistribution

**Fig 7:**
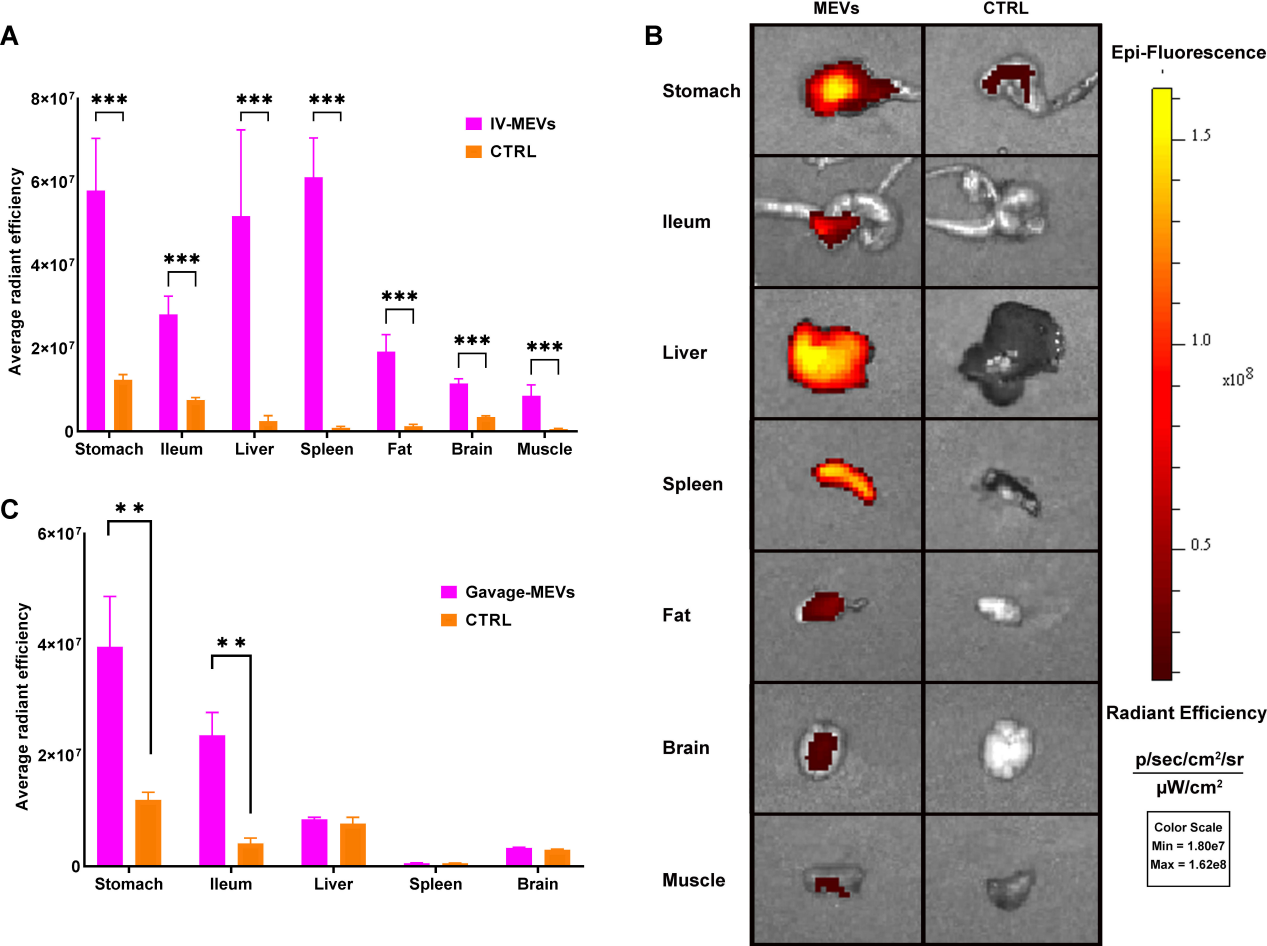
Biodistribution of MEVs in C57BL/6 mice. (**A and B**) Average radiant efficiency of various organs following injection (**A**) or oral gavage (**B**) of Cy7-labeled MEVs compared to control. Asterisks indicate statistical significance (**P < 0.01, ***P < 0.001). Fluorescence imaging (IVIS Lumina XR) of different organs following intravenous injection of MEVs.

In order to assess the MEVs biodistribution in different host organs, Cy7-labeled MEVs were administered IV or by oral gavage to C57BL/6 mice. The fluorescence intensity, expressed as average radiant efficiency, exhibited a significant increase in the stomach, ileum, liver, spleen, fat, brain, and muscles of C57BL/6 mice following intravenous injection with Cy7-MEVs, in comparison to the control mice receiving phosphate-buffered saline (PBS) (Fig. 7A and B). After a 6-h period of gavage with a single dose of MEVs, the fluorescence was detected only in the stomach and ileum but not in other organs (Fig. 7C). These findings suggest that 6 h may not be sufficient to detect MEVs outside the gastrointestinal tract, indicating the need for an extended monitoring period (12 and 24 h) post-gavage administration.

## DISCUSSION

Dysbiosis of the intestinal microenvironment has been linked to many health disorders, including mental and behavioral disorders (4–6). Microbiome-based approaches showed promises as potential disease-modulating strategies (7–12). However, the mechanism by which gut microbiome contributes to these disorders are still hypothetical. In order to achieve a precise modulation of the gut microbiome with clinical effectiveness, it is essential to identify the regulatory mechanisms that control the host-microbiome interactions. Recently, MEVs have been proposed as a key delivery mechanism in controlling intestinal microenvironment and bacteria-host communications (13, 26). However, the production and role of MEVs released by probiotic and commensal microbes in the gut environment are poorly investigated (13, 16), being mostly examined in pathogenic strains (15, 17, 18). Therefore, this study aimed to characterize the cargo of the microbiome-generated EVs and to assess their transport across host barriers and biodistribution to different host organs including the brain. This study was conducted on EVs isolated from human donor fecal samples. However, fecal samples contain EVs generated by both microbiome and host cells. Hence, we developed the gut microbiome of this fecal samples *ex vivo* in a right colon simulator as described before (27), in order to focus only on the microbiome-generated EVs (MEVs). The *ex vivo*-developed microbiome structure was slightly different from that of stool samples as expected because of two reasons, first is enrichment of some low abundant species and depletion of few uncultured species by the effect of the fermentation medium (27). Also, stool samples are known to exhibit different profiles of gut microbiota compared to the proximal colon (28, 29). Still, the *ex vivo*-developed microbiome was of comparable diversity and structure to the colon microbiome of healthy individuals (28).

Our findings underscore the capability of MEVs to traverse and be endocytosed by both intestinal and BBB cell lines. MEVs hold the potential to cross intestinal barriers, thus reaching distant organs like the liver and adipose tissues, consequently possibly instigating insulin resistance and glucose intolerance (30). We observed that MEVs significantly lowered the TEER in Caco-2 cells following exposure to MEVs. This may explain a reported increased level of systemic bacterial EVs in humans with intestinal barrier dysfunction, which provides evidence of MEVs’ capacity to reach the systemic circulation (31). The results of RNA-seq analysis disclosed that the significant reduction in TEER is not due to a dysfunction in tight junction proteins but may be attributable to the modulation of some immune response pathways. These findings are consistent with recently reported results by Mandelbaum et al. (32), where they observed immune response modulation and anti-inflammatory effects of *Bifidobacterium longum* EVs (32). We recently found that *B. finegoldii* EVs induced the expression of IL-6, IL-1β, interferon gamma (IFNγ), and transforming growth factor beta 1 (TGF-β1) in RAW macrophages (33). In agreement with this, the EVs of a well-studied probiotic, *Limosilactobacillus reuteri* DSM 17938, has been shown to induce the expressions IL-6 and IL-1β in peripheral blood mononuclear cells (34). This was explained by the abundance of 5´-nucleotidase (5´NT) in the EVs which is also detected by our recent work as EVs component (33). 5´NT is an enzyme that converts AMP into adenosine, the signaling molecule (34). MEVs content of LPS induces inflammation through toll-like receptor 4 (TLR-4) stimulation, and also, TLR-2 recognizes different molecular patterns including lipoproteins and glycolipid; all are components of MEVs (35). On the other hand, MEVs could also modulate inflammation and cytokine levels through short-chain fatty acids (SCFAs). Price and coauthors reported that intestinal *Bacteroides* modulates inflammation, systemic cytokines levels, and gut microbial ecology through propionate production (36). Also, GABA is known to stimulate GABA_A_ receptors which suppresses T1 response, NF-κB activation, and proinflammatory cytokine production, while stimulating IL-10 signaling (37, 38). Beyond GABA, certain microbiota strains such as *Alistipes putredinis* exert their anti-inflammatory effects through TLR-2 signaling and induction of IL-10 production (39). Similarly, *Bacteroides fragilis* attenuates DSS-induced colitis in mice through the TLR-2/IL-10 signal pathway (40). This effect of EVs on gut immune homeostasis is strain specific as we discussed before in detail (13). Previous reports have shown that EVs released by specific bacteria exhibit different routes of crossing host barriers and induction of variable host response (41, 42). Therefore, the heterogeneous nature of MEVs may explain the employment of different transmission routes across host barriers. For example, EVs from probiotics have been reported to modulate the intestinal barrier integrity and tight junction proteins expression and subcellular distribution through TLR-2 stimulation (43). At the brain level, EVs secreted by *Aggregatibacter actinomycetemcomitans* have been detected in the brain monocytes and microglial cells, and their cargo of RNA has induced neuroinflammation (44). On the other hand, the EVs from *Bacteroides thetaiotaomicron* were illustrated to cross the gut epithelial and blood-brain barriers and to be uptaken by microglia and immature neuronal cells without induction of significant inflammation (45). A congruent study elucidated that *Bacteroides* LPS silences TLR-4 signaling and immunoinhibitory in all healthy adults, indicating that commensal EVs play a role in gut homeostasis (46). Collectively, these findings indicate that MEVs employ different pathways to cross the host barriers, including intestinal and blood-brain barriers, to reach distant organs where they may release their cargo that modulates the organ functionality in a strain-specific way.

MEVs were capable of crossing the hCMEC/D3 human brain endothelial cell line as a model of human blood-brain barrier paracellular permeability (47). *B. thetaiotaomicron*-derived EVs were recently reported to transmigrate across intestinal epithelial and BBB endothelial cell lines (45). Additionally, MEVs were detected in different organs, including the brain, liver, stomach, and spleen, following intravenous injection of Cy7-labeled MEVs in C57BL/6 mice. Our results also showed that 6-h post-gavage administration of MEVs (single dose) was not sufficient for MEVs to be detected in organs outside the gastrointestinal tract (GIT). Longer monitoring time and frequent gavage administration are still required to check the capability of MEVs to cross to the circulation post-gavage administration. Previously, lipophilic EVs, when orally gavaged or injected intravenously or intraperitoneally, were detected in different organs, including the brain (48). This indicates that MEVs that reach the blood circulation can cross the BBB and deliver their cargo to the brain, thus modulating their functionalities. For instance, *Lactiplantibacillus plantarum*-secreted EVs have been reported to suppress stress-induced reduced hippocampal expression of pro-brain-derived neurotrophic factor (BDNF) and BDNF in chronic restraint stress-treated mice (49). The same study postulated that *L. plantarum* EVs injected intraperitoneally may reach the brain and induce direct genomic changes in the brain cells (49).

**Fig 8:**
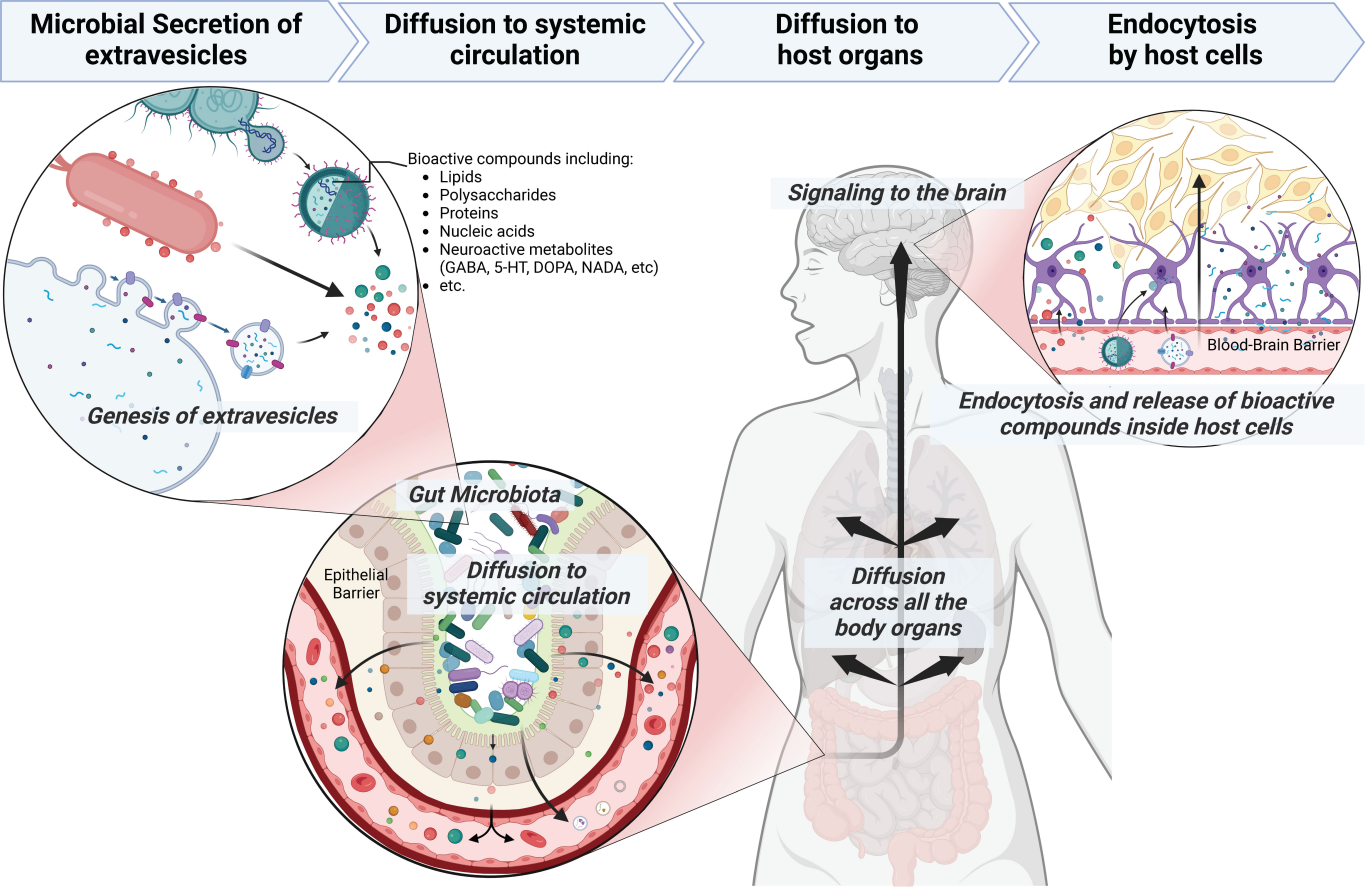
Proposed pathway mechanism of microbiota-released extracellular vesicles in mediating host-microbiome communication and transporting bioactive metabolites.

Our study revealed the presence of a wide range of metabolites embedded in MEVs, including carbohydrates, amino acids, vitamins, and other ionizable molecules. Interestingly, we identified many neurotransmitter-related compounds or their precursors inside MEVs, including arachidonyl-dopamine (NADA), gabapentin, and N-acylethanolamines. Dopamine, a representative neurotransmitter in humans, was found in the gut microbiota-derived MEVs in a conjugated form with arachidonic acid. N-acylethanolamines, such as palmitoyl-ethanolamide and linoleoyl-ethanolamide, have been reported as effective neuroprotective agents (50, 51). Also, NADA is an endocannabinoid with widespread physiological and pharmacological activities, including modulation of neuropathic pain, inflammatory hyperalgesia, and immune and vascular systems (52). Also, several lipid species detected in MEVs are conjugated with HUFAs, such as linoleic acid and arachidonic acid. HUFAs are key molecules for the development, maintenance, and performance of the nervous system as well as brain functionality through improving the fluidity of the cell membrane (53). Collectively, this indicates that MEVs contain neurotransmitter-like molecules proposing MEVs as a signaling shuttle in the gut microbiota-brain axis (Fig. 8).

Gut microbiota is known to impact the level of neuroactive compounds in the gut, periphery, and the brain, and in turn affects the mental status and behavior (54). For instance, colonization of the mice gut with *Akkermansia* and *Parabacteroides* spp. has been reported to protect from seizures through increase of GABA/glutamate ratio in the mice hippocampus (55). The dual presence of these two genera and the metabolic cross-feeding between them was essential to modulate the glutamine/glutamate/GABA pathway, leading to increased GABA/glutamate ratio and restricting the periphera gamma-glutamyl amino acids (55). Here, we show that *B. finegoldii* produces more GABA compared to *P. massiliensis,* while more glutamate is found in the supernatant/EVs of the latter species. However, the cross-feeding between these species and other microbiota members needs to be studied. Multiple microbiota genera possess the ability to either produce or consume GABA or glutamate. Notably, some newly uncultured bacteria have been found to utilize GABA as their sole carbon source, underscoring the multifunctionality of GABA within the gut ecosystem (56). Still, how the gut microbiota-generated GABA, glutamate, and even glutamine reach the brain is unknown. Our findings show that high GABA-producing *B. finegoldii* packs GABA in EVs compared to low GABA-producing *Bacteroides* spp. In accordance with this, a previous report showed that EVs released by *Bacteroides fragilis* include GABA and its intermediates α-ketoglutarate and glutamate as part of their content (57). The oral administration of *B. fragilis* reduced gut permeability, microbiome dysbiosis, and several behavioral abnormalities in a mouse model of autism spectrum disorder, thus highlighting the potential of microbial interventions in modulating gut microbiome-mediated neurological disorders (58). Also, *Bacteroides*, a major GABA-producing genus in the gut, was linked with higher levels of serotonin and myoinositol, which is pivotal in maintaining signaling between the enteric and central nervous systems (59). The relative abundance of *Bacteroides* was negatively correlated with depression-associated brain signatures (56), indicating a significant role of microbiome-secreted GABA in brain functionality. Likewise, Mason et al. (60) have reported depletion of *Bacteroides* in depression and anxiety. Nevertheless, the blood-brain barrier is very selective and will less likely allow extracellular GABA to pass through (61). Extracellular GABA within the gut has multiple other functions acting as carbon source for other bacteria (56), help in acid resistance (62), as well as GABA can cross the epithelial barrier via amino acid transporters and interact with immune system (63). On the other hand, EVs offer advantages over free circulating metabolites and other microbe-associated molecules. For instance, the lipid nature of EVs facilitates their uptake and processing by host cells, and enable them to cross biological barriers such as intestinal barrier and blood-brain barrier (64, 65). The EVs, as nanosized particles, are ideal for intercellular communication and their cargo transport (13). Also, EVs protect their cargo from enzymatic degradation, ensuring the stability of their content, and have the potential to serve as delivery system for targeted drug and biomarkers in different health disorders (66). Together, these findings illustrate the potential of *Bacteroides* spp. and their released EVs as next-generation psychobiotics in different mental disorders. More studies are still required to verify the ameliorative effect of MEVs on neurological and behavior disorders using their animal models.

In addition to delivering the neurometabolites to the central nervous system, neurochemicals, such as GABA, serotonin, dopamine, or their precursors and derivatives, are microbially metabolized by gut commensals and being considered major modulators of the gut environment, including the enteric nervous system (67). We found DOMA, a metabolite of norepinephrine, as a part of MEVs’ content. Sule et al. (68) have reported that DOMA is produced by the metabolic activity of *Escherichia coli*. Also, *Bifidobacterium dentium*, a GABA-producing bacterium, modulated sensory neuron activity in a rat fecal retention model of visceral hypersensitivity (69). Besides, GABA was detected in the cytoplasm and brush border of epithelial cells in the rat jejunum and colon (70). The exposure of GABA to epithelial cells selectively stimulated *MUC1* expression in isolated pig jejunum (71) and increased the expression of tight junctions and TGF-β (72), while decreasing IL-1β-mediated inflammation *in vitro* (72), providing a protective effect against the disruption of the intestinal barrier. Importantly, GABA has also been identified as an essential growth factor that solely can induce the growth of unculturable gut microbes (56). Together, these findings indicate that the metabolites embedded in MEVs may also modulate the gut microenvironment.

In summary, our study provides significant insights into MEVs’ capacity to transfer neuroactive metabolites to the host intestine and other organs, including the brain, filling some of the gaps in knowledge of the mechanisms underlying microbiome-gut-brain interactions. Future research is still needed to understand how MEVs affect the functionality of these organs and identify specific molecular components of MEVs that affect their function.

## MATERIALS AND METHODS

### Isolation of MEVs from stool and *ex vivo*-developed microbiota

Fresh stool samples were collected from 12 healthy donors (*n* = 6 males and 6 females, Table S14). The donors have not received antibiotics or probiotic supplementation for at least 3 months before the donation. The microbiota communities from healthy donors were developed using an *ex vivo* model mimicking the large intestine (27). Immobilization of microbiota was done by inoculating fecal samples into gellan (2.5%, wt/vol) and xanthan (0.25%, wt/vol) gum beads under anaerobic conditions, as described previously (27). Gel beads (30%) were transferred into a stirred glass reactor containing fresh MacFarlane culture medium. The fermentation conditions have been described previously (27). A total of 100 mL of the bioreactor culture after 15 days post-inoculation (microbiota stabilization period) was centrifuged at 14,000 × *g* for 30 min at 4°C. The supernatant was filter-sterilized through a 0.22 µm filter. MEVs were then isolated from the sterile supernatant by ultracentrifugation at 100,000 × *g* for 70 min. MEVs size was determined using an Exoid instrument (Izon Science, MA), which relies on tunable resistive pulse sensing technology. The morphology of the isolated MEVs was inspected by TEM as described previously (23). The isolated MEVs in PBS were diluted 1:100 and fixed in 2.5% glutaraldehyde in 0.1 M sodium cacodylate buffer. The pellets were vortexed thoroughly, and 10 µL of the suspension was added to a carbon grid. Five minutes later, the grid was washed with a drop of distilled water. After drying, 10 µL Uranylless stain was added to the grid and incubated for 1 min before removing excess stain and drying at room temperature (RT) before imaging with TEM.

### DNA extraction for microbiome characterization through 16S rRNA sequencing

DNA was extracted from stool or a 2 mL sample of the 15-day-old *ex vivo* microbiome using the FastDNA Spin Kit (MO BIO Laboratories Inc.) according to the manufacturer’s instructions, with the addition of an extra cycle of mechanical homogenization and 5 min of cooling on ice between the two cycles. The extracted DNA was quantified using a Qubit fluorometer and Qubit dsDNA, broad range, quantification assay kit (Invitrogen, Carlsbad, CA, USA) and stored at −20°C until use.

### DNA library preparation for 16S rRNA gene sequencing

The microbiome diversity was determined by sequencing the V3-V4 regions of the 16S rRNA gene using the Illumina MiSeq platform and MiSeq Reagent Kit v.3 (600-cycle), and by employing Illumina standard protocol, as described before (27). The raw paired-end sequences were fed to the Quantitative Insights Into Microbial Ecology 2 for quality preprocessing and determination of microbial composition and diversity indices (73). Briefly, the imported sequences were denoised using DADA2 plugin (74). The denoised reads were then rarified to the depth of 11,000 reads per sample; two samples (D7F and D11S) did not have enough sequences for the rarefaction depth, so they were excluded from further analyses. The rarified reads were then used for core metrics phylogenetic diversity analyses. Alpha-diversity was calculated and presented as Shannon index. Beta-diversity and principal coordinate analyses were conducted based on Bray-Curtis distances. Permutational multivariate analysis of variance using Adonis was used for statistic analyses of beta-diversity results. Taxonomy was assigned to the generated features using scikit-learn naive Bayes machine-learning classifier trained on Silva 138 99% operational taxonomic units (OTUs) full-length sequences (75–77). Linear mixed model was used for statistical comparisons between stool and *ex vivo* predominant microbiota features (prevalent in more than 50% of samples), using donor as confounding variable.

### Extraction of metabolites from MEVs

A cold methanol extraction method was used to extract metabolites from extracellular vesicles. Briefly, 900 µL of cold MS-grade methanol was added to 100 µL of the extracted EVs. Ten microliters of 13C-phenylalanine (1 mg/mL) or 1 µL of 1 µg/mL reserpine solution was added to the mix as an internal standard. The sample was vigorously vortexed for 1 min and kept at −20°C for 15 min. The sample was thawed for 3 min at room temperature and then vigorously vortexed for 1 min again. The resulting sample was centrifuged for 5 min at 12,000 × *g* at 4°C. The supernatant was vacuum dried using SpeedVac concentrator. The dry extract was dissolved in 100 µL of a mixture consisting of 50% acetonitrile, 50% water, 0.1% formic acid (positive mode) or 1 mM ammonium acetate (negative mode). The sample was passed through a 0.2 µm polytetrafluoroethylene (PTFE) filter and was analyzed immediately. The calibration curve of GABA and GA were conducted using the following concentrations of compounds: 10, 100, 250, 500, 750, and 1,000 µM.

### nLC-nESI-MS/MS metabolomics analysis

The extracted metabolites from the EVs were analyzed by nanoLC coupled to the Q-Exactive Plus mass spectrometer (Thermo Fisher Scientific). Chromatographic separation of metabolites was performed on a Proxeon EASY nLC II System (Thermo Fisher Scientific) equipped with a Thermo Scientific Acclaim PepMap RSLC C18 column (P/N ES800A), 15 cm × 75 µm ID, 3 µm, 100 Å, employing a water/acetonitrile/0.1% formic acid gradient. One milliliter of samples was loaded onto the column for 60 min at a flow rate of 0.25 µL/min. Compounds were separated using a linear gradient from 0 to 100% of acetonitrile for 35 min, followed by washing for 10 min at 100% of acetonitrile, then using a gradient from 100 to 0% of acetonitrile for 5 min and washing for 10 min at 100% of water. Eluted compounds were directly sprayed into the mass spectrometer using either positive or negative electrospray ionization (ESI) at an ion source temperature of 250°C and an ionspray (Thermo Scientific EASY spray) voltage of 2.1 kV. The fourier transform mass spectrometry (FTMS) scan type was full MS/data-dependent (dd)-MS2. The parameters of the full mass scan were as follows: a resolution of 70,000, an auto gain control target under 3 × 10^6^, a maximum isolation time of 100 ms, and an m/z range of 100–1,000. The parameters of the dd-MS2 scan were as follows: a resolution of 17,500, an auto gain control target under 1 × 10^5^, a maximum isolation time of 100 ms, a loop count of top 10 peaks, an isolation window of m/z 2, charge exclusion of 3–8, > 8, a normalized collision energy of 35, and dynamic exclusion duration of 10 s. The LC-FTMS system was controlled using Xcalibur 4 software (Thermo Fisher Scientific), and data were collected with the same software.

### nLC-nESI-MS/MS metabolomics data processing and analysis

Mass spectrometry data were processed and analyzed by MS dial v.4.60 along with the Massbank and Human Metabolome Database to facilitate the identification of target molecules (78). Default parameters were used to identify individual compounds in samples, and contaminants detected in the blank were excluded from the list of identified compounds.

### Preparation of samples for nano-LC-MS/MS proteomics analysis

Protein concentrations were measured using the Bradford protein assay, and 50 µg of protein was processed using a filter-aided sample preparation protocol. Exosomal fractions of approximately 50 mg were adjusted to 50 mM TRIS, 8 M urea, 5% (vol/vol) glycerol, and either 0.1% n-dodecyl-β-D-maltoside (DDM) or SDS (pH 8.0) and vortexed for 30 s. The suspension was centrifuged for 5 min at 10,000 × *g*, and the supernatant was separated from the insoluble debris and passed through a 10 kDa molecular weight cut-off (MWCO) filter (Millipore). Sample volumes were reduced to approximately 20 µL by vacuum centrifugation for 25 min at 14,000 × *g*, and proteins were reduced by the addition of 4 mM tris(2-carboxyethyl)phosphine (TCEP) in 100 µL denaturation buffer for 45 min (30 min incubation + 15 min centrifugation) at RT. Buffer was exchanged to alkylate proteins with 20 mM iodoacetamide in 100 µL denaturation buffer for 1 h at RT in the dark. The filter was washed once with digestion buffer (50 mM TRIS, 0.6% glycerol, pH 8) before being transferred to a clean collection tube. Digestion was performed with 300 ng trypsin/Lys-C (Promega, V5072) in 100 µL of digestion buffer at 37°C for 12 h. Peptides were eluted from the filter by centrifugation at 14,000 × *g* for 10 min, and an additional centrifugation step was performed after adding 40 µL digestion buffer. Eluted peptides were treated with formic acid at a final concentration of 1%. The peptides were desalted on TopTip C-18 microspin columns (Glygen, # TT2C18) and dried by vacuum centrifugation. Samples were analyzed using an Orbitrap Fusion mass spectrometer (Thermo Fisher Scientific, Mississauga, ON, Canada) coupled to an UltiMate 3000 nanoRSLC (Thermo Fisher Scientific, Mississauga, ON, Canada), as described previously (79). The mass spectrometry proteomics data have been deposited to the ProteomeXchange Consortium (80) via the PRIDE (81) partner repository with the data set identifier PXD044889.

### Protein identification, quantification, and profiles

Peptide/protein identification and quantification, peptide taxonomic assignment, and functional protein annotation were performed using iMetaLab software v.2.3 (25) using a database based on the integrated gene catalog, which contains a complete set of genes for gut microbiota members (82). Therefore, we did not identify human protein markers. This also could be the reason why we get a higher number of proteins identified in MEVs from fermenter samples than those of stool samples, as stool sample is a mixture of eukaryotic and microbiota EVs. Hence, we just did a qualitative description of their content without quantitative comparison. We only compared the function within each population and not between the populations. We employed the MaxQuant search engine in the MetaLab workflow for peptide/protein identification (83) (Table S15). Carbamidomethyl (C) was set as a fixed modification, and protein N-terminal acetylation (protein N-term) and oxidation (M) were set as variable modifications. Peptide and protein identification was conducted with a false discovery rate of 0.01. Functional protein annotation was performed using COG and KEGG Orthology databases.

### Isolation and characterization of commensals with psychobiotic activity

#### Bacterial strains isolation

To ensure accurate and representative analysis, the fecal sample was subjected to a thorough homogenization process using reduced peptone water (2%, wt/vol). Subsequently, serial dilutions ranging from 10^−1^ to 10^−8^ were prepared, and the diluted samples were spread on fastidious anaerobic agar with 0.5% yeast extract and brain heart infusion with yeast extract, cysteine, and hemin. The agar plates were meticulously maintained under strictly anaerobic conditions comprising 5% hydrogen, 10% CO_2_, and 85% nitrogen for 5 days to facilitate optimal microbial growth. Following incubation, individual colonies were selected and streaked onto their respective agar plates to ensure the purity of the isolates. Single colonies from each plate were then introduced into a culture broth, allowing for their preservation in 25% glycerol at an ultralow temperature of −80°C.

### GAD assay

For molecular identification of GABA-producing bacterial isolates, an initial screening was conducted using the GAD assay (84). A hypertonic test substrate solution was prepared by combining 0.1 g of L-glutamic acid, 30 µL of Triton X-100, 9 g of NaCl, and 0.005 g of bromocresol green as an indicator in 100 mL of sterilized water, adjusted to a pH of 4.0, resulting in a yellow-colored solution. After centrifuging the bacterial culture (grown for 48 h), the pellet was washed with 0.9% saline, and 0.3 mL of the test solution was added to the washed pellet. A change in color from yellow to green/blue was used to indicate the potential GAD activity.

### DNA extraction, 16S rDNA-based characterization, and taxonomic assignment

DNA extraction from the bacterial isolates was carried out using the NucleoSpin Microbial DNA kit (Macherey-Nagel, Duren, Germany) following the manufacturer’s instructions. To amplify the 16S rRNA gene, PCR was performed utilizing the widely recognized universal primers Bact8F and 1391R (84). The PCR reaction mixture comprised 1× PCR buffer, 1.5 mM MgCl_2_, 0.2 mM nucleoside triphosphates (NTPs) (Invitrogen, Carlsbad, CA, USA), 1 µM of each primer, 1U of Taq DNA polymerase (Invitrogen), and 50–100 ng of bacterial DNA, in a total volume of 50 µL of nuclease-free water. The PCR amplification protocol consisted of an initial denaturation step at 95°C for 3 min, followed by 30 cycles of denaturation at 95°C for 30 s, annealing at 55°C for 30 s, extension at 72°C for 60 s, and a final extension step at 72°C for 5 min. Successful amplification of the PCR products was confirmed by gel electrophoresis on a 1.2% agarose gel containing ethidium bromide. The PCR products were subsequently purified using a QIAquick PCR purification kit (Qiagen, Hilden, Germany) and sent for Sanger sequencing at the Ottawa Hospital Research Institute’s DNA sequencing facility (Ottawa, ON, Canada). The chromatograms obtained were subjected to quality assessment using BioEdit. Taxonomic classification of the sequences was performed using the Ribosomal Database Project (RDP) classifier (85), and a nucleotide similarity threshold of 99% (16S rRNA gene) was employed to confidently identify the isolates at the species level (86). The taxonomic identification was validated by Basic Local Alignment Search Tool (87) against the 16S ribosomal RNA database.

### Whole-genome sequencing, annotation, and comparative genome analysis

A Nextera DNA Flex kit (Illumina) was used to prepare DNA libraries following the Illumina recommended protocol. Illumina paired-end (2  ×  151 bp) whole-genome sequencing data were obtained using MiSeq. The raw reads were demultiplexed and subjected to sequencing adapter trimming using MiSeq Local Run Manager v.3 (Illumina). Subsequently, the reads underwent quality and length filtering using the FASTQ Toolkit v.2.2.5. The Illumina reads were *de novo* assembled using Velvet Assembler v.1.0.0 integrated into the BaseSpace Sequence Hub (Illumina). For annotation of the assembled contigs, the Rapid Annotations using Subsystem Technology Server and automated NCBI PGAP annotation were utilized (88, 89).

To unravel the intricacies of the GAD system across various *Bacteroides* species, we conducted a comprehensive binning of whole genomes. This approach provided valuable insights into the structural aspects of the GAD system within these species. Additionally, to gain a deeper understanding of *Bacteroides* capacity to utilize complex glycans, we employed PULpy software to perform automated prediction and analysis of the number and structure of polysaccharide utilization loci within each genome (90).

### Phylogenetic analysis

The phylogenetic trees for *gadA* and *gadB* were constructed using IQ-TREE release 2.2.2.6 (91) (http://www.iqtree.org/). The best-fit model according to Bayesian information criterion was determined using the built-in ModelFinder within IQ-TREE, resulting in TNe + G4 and TIM2e + G4 as best-fit models for *gadA* and *gadB* genes, respectively. The resulting trees were visualized using Interactive Tree Of Life version 6 (https://itol.embl.de/). Bootstrapping was performed with 1,000 replicates, and the values are displayed as circles alongside the branches, representing the proportion of trees in which associated taxa formed distinct clusters.

### ELISA tests for quantification of GABA

We employed a competitive ELISA approach to quantify GABA in the 18 *Bacteroides* strains using GABA ELISA Kit (Abnova). Initially, single colonies were inoculated into fastidious anaerobic broth (FAB) and allowed to grow for 24 h. Subsequently, the resulting preculture was added at a 1.25% (vol/vol) ratio to 8 mL FAB supplemented with 10 mM glutamate (pH 6.5). The cells were incubated under anaerobic conditions at 37°C for 72 h. To prepare the samples for analysis, 2 mL of bacterial cultures were centrifuged at 14,000 × *g* for 10 min at 4°C. The resulting supernatants were carefully collected, filtered through a 0.22 µm filter, and stored at −20°C until further use in ELISA assays. GABA quantification was performed in three independent replicates using an ELISA kit (Abnova, Taipei, Taiwan), following the manufacturer’s protocols to ensure accurate and reproducible measurements of GABA levels in the samples.

### Purification of EVs from bacterial isolates and metabolite and proteome extraction

We conducted comparative metabolite profiling to elucidate the metabolites released by high and low GABA producers and investigate the presence of neurotransmitter-related compounds within EVs. A 120 mL culture of *B. finegoldii* UO.H1052 or *P. massiliensis* UO.H1001 grown for 72 h was centrifuged at 15,000 × *g* at 4°C for 30 min. The resulting supernatant was filtered through a 0.22 µm filter, followed by ultracentrifugation at 100,000 × *g* for 1 h using an Optima L-90K ultracentrifuge (Beckman Coulter). The supernatant was carefully stored at −20°C, and the pellet was washed with 5 mL of sterile PBS, followed by an additional round of ultracentrifugation. The vesicle pellet was then resuspended in distilled water.

For metabolite extraction, 100 µL of the resuspended EVs were combined with 900 µL of methanol (−20°C) and incubated at −80°C for 15 min. The sample was thawed for 3 min at room temperature, vigorously vortexed, and centrifuged for 30 min at 16,000 × *g* at 4°C. The supernatant was subsequently vacuum-dried using a SpeedVac concentrator (Thermo Fisher Scientific, USA). The resulting dried extract was dissolved in 100 µL of sterile water and subjected to GABA quantification along with the supernatant using an ELISA method. Proteins and metabolites in EVs were subjected to proteomics and metabolomics as aforementioned.

### Cell culture experiments

All reagents were purchased from MilliporeSigma (Toronto, ON) unless otherwise specified. Dulbecco’s Modified Eagle Medium (DMEM), RPMI-1640 media, L-glutamine, penicillin/streptomycin, chemically defined lipid concentrate, HEPES, trypsin-ethylenediaminetetraacetic acid (trypsin-EDTA), trypan blue, Dulbecco’s PBS (DPBS) were purchased from Thermo Fisher Scientific (Gibco, Nepean, ON). Tissue culture well plates (12, 24, and 48), flasks (25 and 150 cm^2^), and inserts (0.4 µm, 12-well plates) were purchased from Corning (Maine, USA). Tissue culture inserts, 0.4 µm (12-well plates), were purchased from ThinCert, Greiner Bio-One, Monroe, NC. Rat collagen I and recombinant human basic fibroblast growth factor were purchased from R&D Systems (Toronto, ON). Endothelial cell growth basal medium-2 (EBM-2) was purchased from Lonza (Kingston, ON).

### Endocytosis activity

RIN-14B cells (ATCC CRL 2059) were grown to confluence in a complete culture medium (CCM) consisting of RPMI-1640 supplemented with 10% heat-inactivated FBS (HI-FBS), 100 U/mL penicillin, and 100 mg/mL streptomycin (92). Caco-2 cells (ATCC HTB 37) were grown to confluence in CCM constituted of DMEM supplemented with 10% HI-FBS, 100 U/mL penicillin, and 100 mg/mL streptomycin (93), while hCMEC/D3 (Cedarlane Labs, CLU 512) were grown on collagen-coated flasks using CCM constituted of EBM-2 supplemented with 5% HI-FBS, 100 U/mL penicillin, 100 mg/mL streptomycin, hydrocortisone (1.4 µM), ascorbic acid (5 µg/mL), chemically defined lipid concentrate (1%), HEPES (10 mM), and basic fibroblast growth factor (1 ng/mL) (94). All cells were maintained at 37°C in a 5% CO_2_ humidified incubator (VWR, Mississauga, Ontario). For subculturing, different cells were dissociated using 0.05% trypsin-EDTA, centrifuged, and washed twice using DPBS (pH 7.4) to remove traces of trypsin (95).

Caco-2 cells at a density of 2 × 10^5^ cells (96) and RIN-14B at 2.5 × 10^5^ cells were seeded in 12-well plates (97). In contrast, hCMEC/D3 cells were seeded at 2 × 10^5^ on a collagen-coated 12-well plate using rat tail collagen (150 µg/mL) (98) and maintained at 37°C in a 5% CO_2_ humidified incubator for 2 days (Caco-2 and hCMEC/D3 cells) and 5 days (RIN-14B) with refreshing media every 3–4 days. MEVs were labeled with Cy7, added to each well of different cells, and incubated for 24 h. Conditioning media containing the unbound MEVs were discarded. Cells grown in the absence of labeled MEVs served as a negative control. The cells were washed three times, mounted in DPBS, and then imaged using an inverted Zeiss AxioObserver 7 microscope (Carl Zeiss Canada Ltd, Toronto, ON).

### Paracellular transport activity

#### Caco-2 cells

Caco-2 cells were seeded at a density of 5 × 10^4^ (P8) onto ThinCert tissue culture inserts (12-well plates, 0.4 µm, 108 pores, 1.131 cm^2^) and then maintained in CCM (99). The culture media were refreshed every 2 days. Cells were allowed to grow and differentiate for 21 days. On day 21, CCM was replaced with PBS, and resistance values (Ω) were measured for all tissue culture inserts, including the blank, using Millicell ERS-II voltohmmeter (MilliporeSigma, Toronto, ON) (100). The TEER values for each insert were calculated according to the following equation:

TEER = (R (Cells) − R (Blank)) × tissue culture inserts surface area (Ω.cm^2^) (100)

MEVs labeled with FITC (101, 102) (equivalent to 5.95e + 11, 2.975e + 11, 1.487e + 11 particles) were suspended in CCM and added to the apical side of the tissue culture insert. We quantitatively measured the LPS associated with MEVs using the limulus amebocyte lysate assay (QCL-1000, Lonza). LPS from *Serratia marcescens* (Sigma) was used as a positive control for the assay. LPS concentrations were 4.62 µg/mL, 10.34 µg/mL, and 20.01 µg/mL, respectively. FITC-dextran (FITC-CM-Dextran 4 [FD-4], M. Wt 4 kDa, MilliporeSigma, cat. number 68059) was used as a positive control of the *in vitro* paracellular permeability of Caco-2 cells (Fig. S11). Fresh CCM was added to the basolateral side, and the conditioned culture supernatant was collected from the basolateral side at 0, 3, and 24 h. The fluorescence intensity was measured using a TECAN plate reader at an excitation/emission wavelength of 485/535 nm. PBS replaced conditioned media in the apical and basolateral compartments, and TEER values were reported for various treatment groups.

#### hCMEC/D3 cells

For the blood-brain barrier transport study, hCMEC/D3 cells were seeded at a density of 5 × 10^4^ onto collagen-coated inserts (12-well plates) (94) using CCM and allowed to differentiate for 4 days. TEER values were recorded every day. The TEER values for each insert were calculated according to the aforementioned equation. After identifying the time point showing the peak TEER value, MEVs labeled with Cy7 (103) (equivalent to 5.95e + 11, 2.975e + 11, 1.487e + 11 particles) were suspended in CCM and added to the apical side of the tissue culture insert (Corning). Fresh CCM was added to the basolateral side, and the conditioned culture supernatant was collected from the basolateral side at 0, 3, and 24 h. The fluorescence intensity was measured using a TECAN plate reader at an excitation/emission wavelength of 743/780 nm. PBS then replaced conditioned media in the apical and basolateral compartments, and TEER values were measured for various treatment groups.

### RNA-seq of Caco-2 cells exposed to MEVS and computational analyses

To decipher the interplay of MEVs with host immune system, we analyzed the modulation in gene expression in Caco-2 cells after exposing them to MEVs for 24 h as aforementioned for transport study in comparison to non-exposed cells. Following exposure, all the media was aspirated, and the Caco-2 monolayer cells were washed with PBS. Subsequently, we extracted total RNA from the epithelial cells using the Trizol method. The extracted RNA was DNase treated and purified using RNeasy MinElute Cleanup Kit (Qiagen) following the manufacturer’s instructions. RNA sequencing was performed at Genome Québec (Montreal, Canada). Briefly, total RNA was quantified, and its integrity was assessed using a LabChip GXII (PerkinElmer) instrument. Libraries were generated from 250 ng of total RNA using Illumina Stranded mRNA Prep, Ligation kit (Illumina), as per the manufacturer’s recommendations. Libraries were quantified using the KAPA Library Quantification Kits - Complete kit (Universal) (Kapa Biosystems). Average size fragment was determined using a LabChip GXII (PerkinElmer) instrument. The libraries were normalized and pooled and then denatured in 0.05N NaOH and neutralized using HT1 buffer. The pool was loaded at 175pM on an Illumina NovaSeq S4 lane using Xp protocol as per the manufacturer’s recommendations. The run was performed for 2 × 100 cycles (paired-end mode). A phiX library was used as a control and mixed with libraries at 1% level. Base calling was performed with RTA v.3.4.4. Program bcl2fastq2 v.2.20 was then used to demultiplex samples and generate fastq reads. The RNA-seq data are now accessible in NCBI’s Gene Expression Omnibus (GEO) database under the GEO series accession number GSE241164.

Analysis was performed using Human GENCODE (104) v.43 annotations based on the GRCh38 genome assembly. The nf-core/rnaseq pipeline version 3.11.2 was used to generate the quality assessment and read pseudocounts table (105). Fold change gene expression was determined using DESeq2 (106). Functional enrichment analysis of the significantly differentially expressed genes was performed using R package, gprofiler2 (107).

### *In vivo* assessment of MEVs biodistribution

To assess the *in vivo* trafficking of MEVs to the systemic circulation and their potential diffusion across the blood-brain barrier, the inbred strain C57BL/6 mice were used as an animal model. A total of 9 C57BL/6 mice (female, 13–15 weeks, 18.9–22.6 g) were used for the different experimental groups. Mice were acclimatized for 3–7 days under ambient conditions (12 h light/ dark cycle at 22 ±2°C and humidity 50-60%) with free access to sterile water and a standard rodent soft chow *ad libitum*. Two distinct types of experiments were meticulously conducted with the mice, involving IV administration in one group and gavage in the other, both incorporating control (CTRL) groups. In the first experimental group (*n* = 6), purified MEVs labeled with Cy7 (2.38e + 11 particles) were administered through the tail vein, while the second group (*n* = 6) received an equivalent number of MEV particles via gavage. Control groups comprised three mice (*n* = 3) and were either injected or gavaged with an equal volume of PBS (placebo control).

Subsequently, the different animal groups were euthanized after intubation using 1–3 isoflurane, with the IV group and its CTRL assessed at 30 min, and the gavage group and its CTRL examined after 6 h. Various tissues, including liver, fat, muscle, spleen, large intestine, and brain, were collected from the animals to perform organ imaging using the IVIS Lumina XR (PerkinElmer, Woodbridge, ON). The excitation/emission wavelengths of 756/779 nm were employed to evaluate the biodistribution of MEVs. The fluorescence intensity of imaged organs collected from both test and control mice was expressed as average radiant efficiency, ensuring accurate quantification and comparison (108, 109).

### Statistical analysis

To compare the mean values of different results, one-way analysis of variance (SPSS, version 23) followed by Tukey’s as a *post hoc* test was used to determine the statistically significant difference for data involving more than two experimental groups. Paired sample *t*-test was used to identify the statistically significant difference between the mean TEER values of Caco-2 and hCMEC/D3 cells before and after MEVs treatment compared to the control. An independent sample *t*-test was used to compare data from the *in vivo* experiments. All results were expressed as mean ± standard error.

## Data Availability

The 16S rRNA gene sequence and genome data of bacterial isolates are available from the NCBI (OP690542-OP690599 and PRJNA898401, respectively). The 16S rRNA metagenomic data are available from NCBI with identifier PRJNA987349. The mass spectrometry proteomics data have been deposited to the ProteomeXchange Consortium via the PRIDE partner repository with the datasetdata set identifiers PXD044889 and PXD045162. Metabolomics data have been deposited to the EMBL-EBI MetaboLights database with the identifier MTBLS8494.

## SUPPLEMENTAL MATERIAL

Supplemental figures spectrum.01368-24-s0001.pdf Fig. S1 to S12.

Supplemental tables spectrum.01368-24-s0002.xlsx Tables S1 to S15.

ASM does not own the copyrights to Supplemental Material that may be linked to, or accessed through, an article. The authors have granted ASM a non-exclusive, world-wide license to publish the Supplemental Material files. Please contact the corresponding author directly for reuse.
