# Supplementary material for: Multi-level analysis of gut microbiome extracellular vesicles-host interaction reveals a connection to gut-brain axis signaling
Source: Microbiol Spectr. 2024 Dec 19;13(2):e01368-24. doi: 10.1128/spectrum.01368-24 (PMC11792502; doi:10.1128/spectrum.01368-24)
Supplement: Supplemental figures — Fig. S1 to S12. [file spectrum.01368-24-s0001.pdf]

Supplemental Figures

Comprehensive multi-omics characterization of gut microbiome extracellular vesicles reveals a connection to gut-brain axis signaling

Walid Mottawea, Basit Yousuf, Salma Sultan, Tamer Ahmed, JuDong Yeo, Nico Hüttmann, Yingxi Li, Nour Elhouda Bouhlel, Heba Hassan, Xu Zhang, Zoran Minic, Riadh Hammami

Supplementary Results

Bacterial strain isolation

A collection of different gut bacterial strains was isolated from a fecal sample obtained from a healthy adult Canadian female donor. The isolated strains demonstrated considerable diversity, with Bacteroidetes emerging as the dominant group, followed by Actinobacteria and Firmicutes. For the specific objectives of this study, we focused on the Bacteroidetes group. We initially selected 58 potential GABA producing isolates through the glutamate decarboxylase (GAD) assay. Afterwards, we conducted a molecular identification using 16S rRNA sequencing. Analysis of the 16S rRNA gene sequences revealed that 28 strains belonged to the *Bacteroides* genus, 22 to *Phocaeicola*, 3 to *Parabacteroides*, and one each of *Butyricimonas* and *Alistipes* (Table S9). In addition, three isolates exhibited a nucleotide similarity of 98.2% with the *P. massiliensis*-type strain in the RDP database and validated in NCBI, indicating the presence of previously undescribed species within the *Bacteroidaceae* family, designated as *Bacteroidaceae* bacterium. From the initial pool of 58 isolates, we selected 18 representative strains for whole-genome sequencing, covering various genera. The selected strains included *Bacteroides cellulosilyticus* UO.H1027 and UO.H1030, *Bacteroides dorei* UO.H1033, *Bacteroides faecis* UO.H1051, *Bacteroides stercoris* UO.H1035, UO.H1039, and UO.H2001, *Bacteroides uniformis* UO.H1043, *Phocaeicola massiliensis* UO.H1001, *Phocaeicola vulgatus* UO.H1015 and UO.H1016, *Bacteroides ovatus* UO.H1053, *Bacteroides finegoldii* UO.H1052, *Bacteroides zhangwenhongii* UO.H1054, and *Bacteroides caccae* UO.H2003, two *Parabacteroides* species (*P. johnsonii* UO.H1047 and UO.H1049) and one unclassified *Bacteroidaceae* bacterium (UO.H1004). In order to assess if the GABA producing capability is related to the sequence similarity of *gadA* and *gadB* genes, we extracted *gadA* and *gadB* gene sequences and clustered the strains based on the similarity of the sequences of both genes (**Figure S12**). We did not observe clustering of the strains based on the GABA productivity (**Figure S12**). *In silico* analysis of the glutamate decarboxylase (GAD) genetic system was performed to investigate the structural diversity among these 13 different *Bacteroides* species. Notably, the *gadC* (glutamate/GABA antiporter-encoding gene) and *gadD* (potassium K+ channel transporter) genes were found in close proximity in most *Bacteroides* strains, suggesting their functional importance. Our *in-silico* analysis revealed the presence of diverse *gadA*/*gadB*/*gadC*/*gadD* genes based on nucleotide similarity amongst the 13 *Bacteroides* species (data not shown).

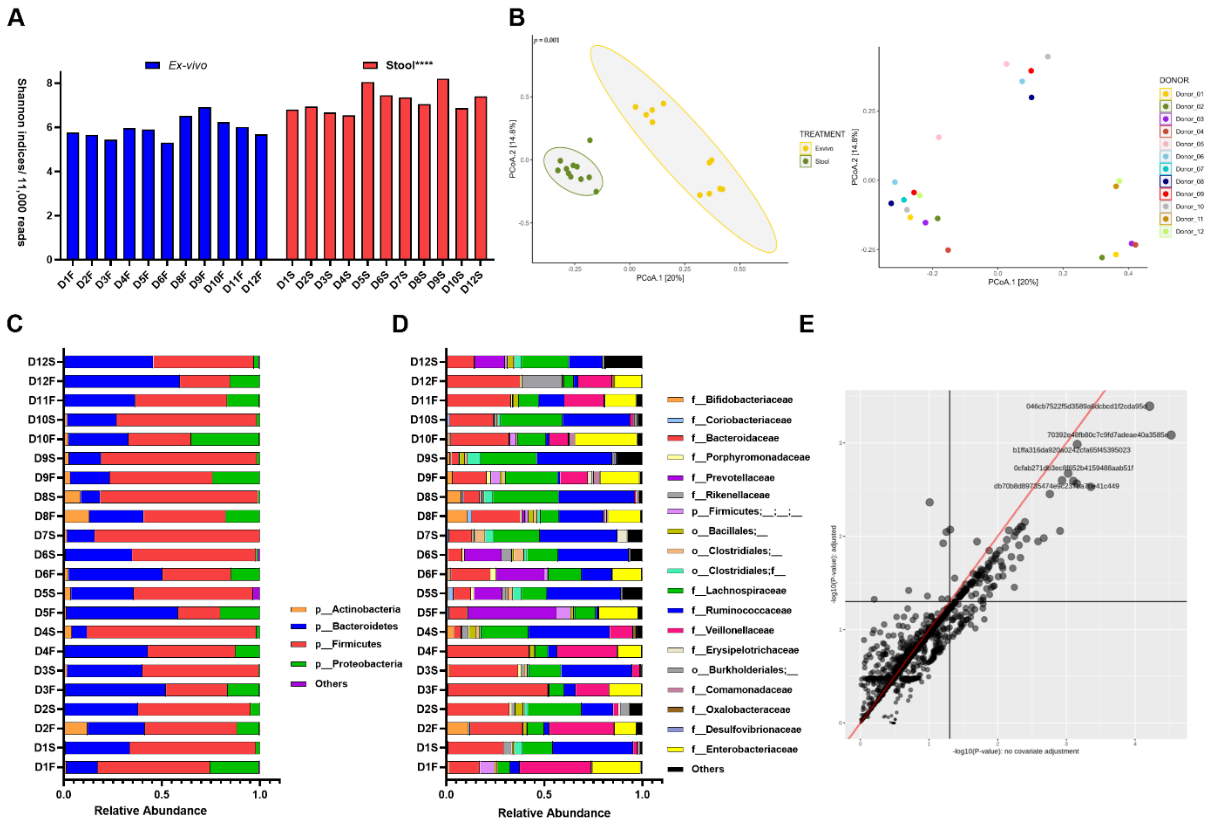

Figure S1. Gut microbiota structure from which MEVs were generated. (A) Diversity of gut microbiota in each sample as indicated by Shannon indices. (B) Principal coordinate analyses of different samples based on the relative abundance of different features identified in each sample generated by QIIME 2.6. Permutational Multivariate Analysis of Variance using Adonis was used for statistic analyses (P<0.001 comparing stool and *ex vivo* samples). (C) and (D) Composition of the gut microbiota at the phyla and family level. (E) Linear model showing the variable features between stool and ex vivo generated microbiome using donor as a covariate for adjustment.

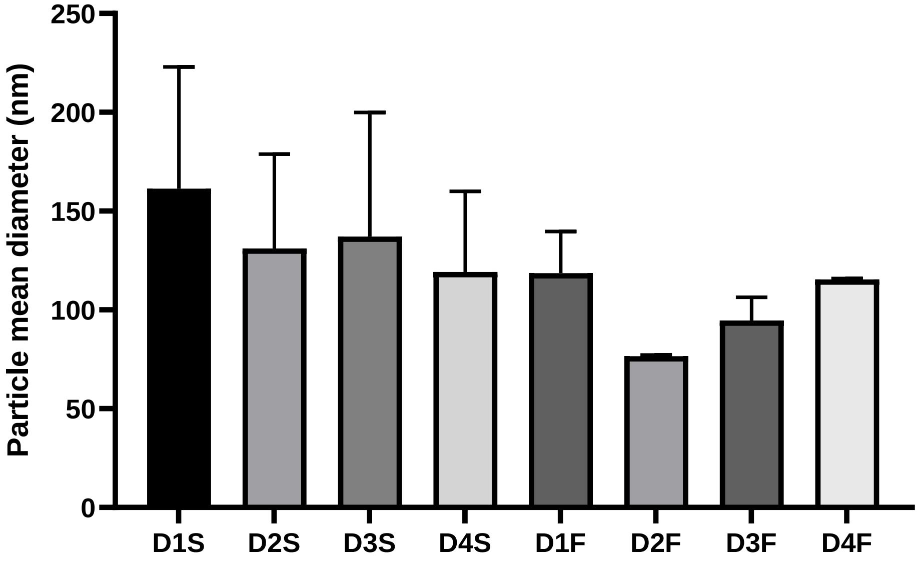

Figure S2. Nanoparticle size analysis of the isolated MEVs using Zetasizer in individual samples.

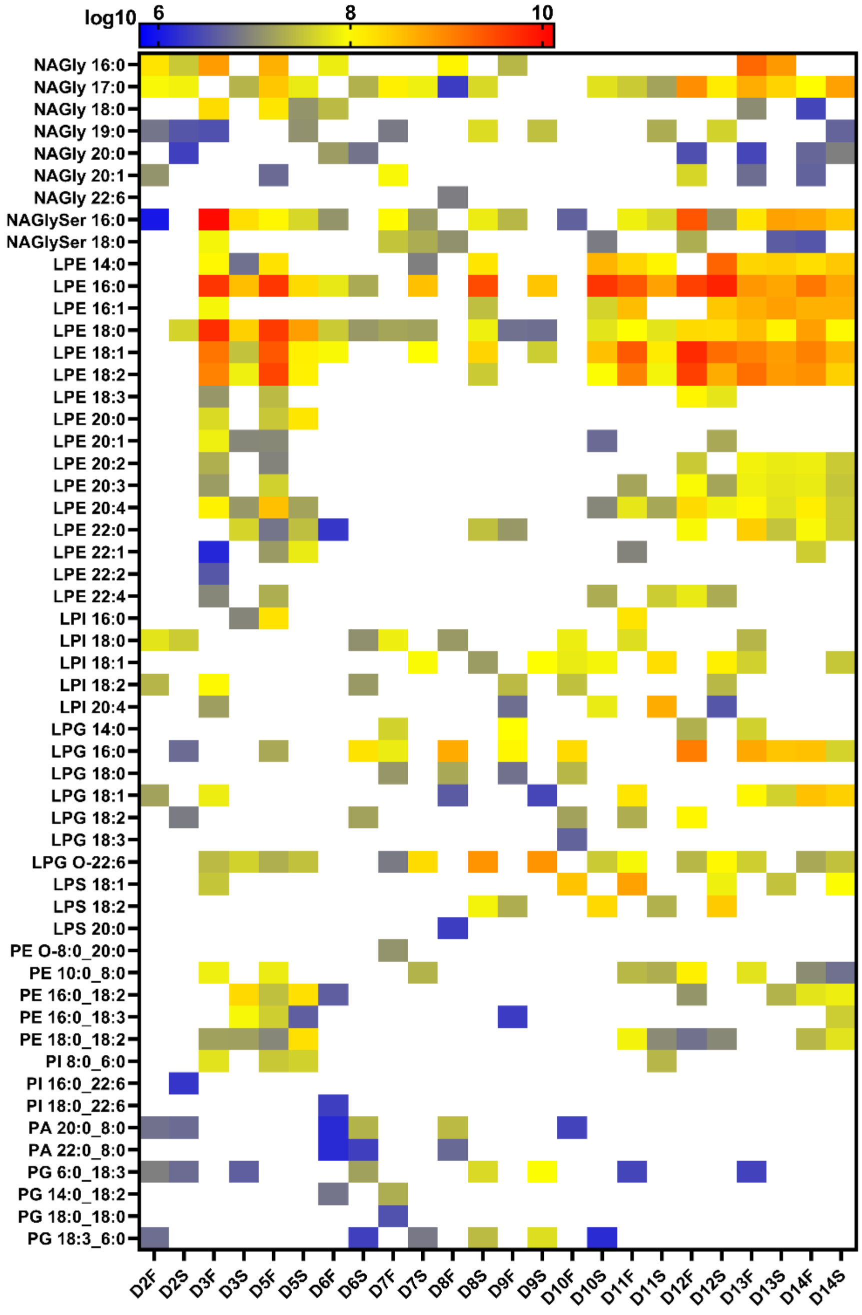

Figure S3. Lipidomics analysis of gut microbiota-derived MEVs conducted <u>in the negative mode</u>. The bar graph illustrates the relative abundance of the individual lipid species identified in the isolated MEVs. NAGly: N-Acetylglycine; NAGlySer: N-acyl glycylserine; PE: Phosphatidylethanolamines; PI: Phosphatidylinositol.

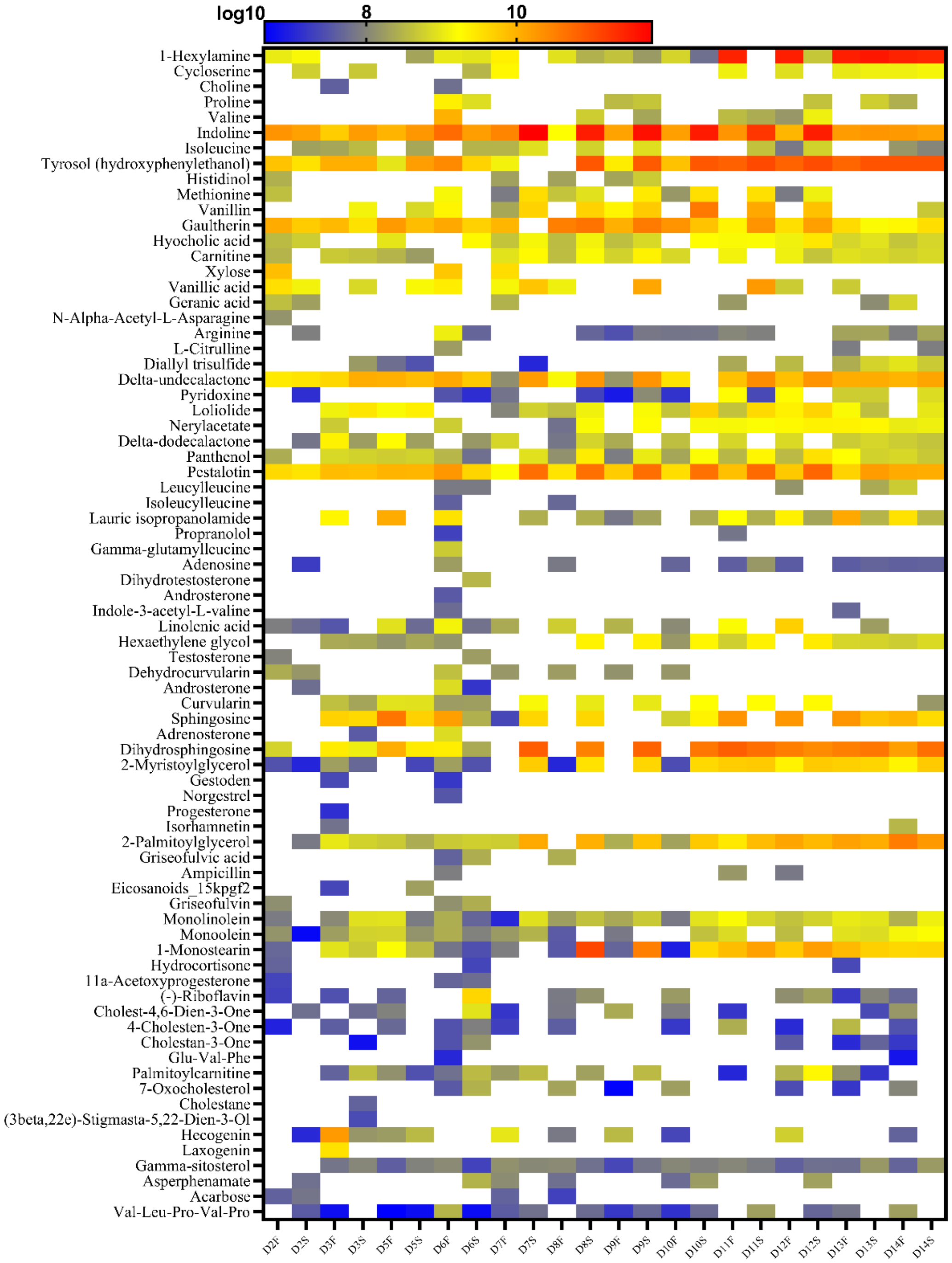

Figure S4. Untargeted metabolomic analysis of MEVs conducted in the positive mode. The heat maps depict the abundance (log10) of individual metabolites identified in the isolated MEVs.

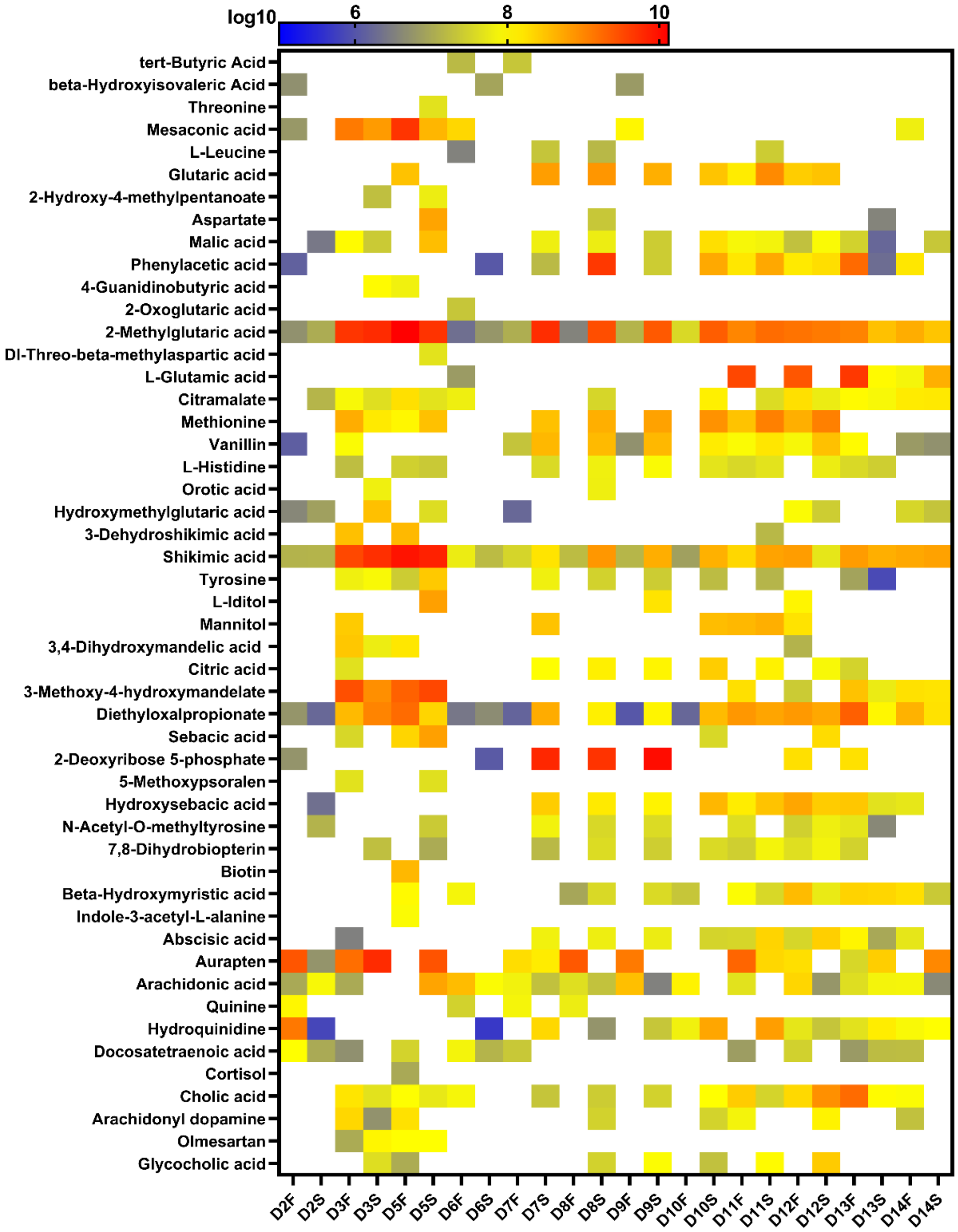

Figure S5. Untargeted metabolomic analysis of MEVs conducted in the <u>negative mode</u>. The bar graph illustrates the relative abundance of the individual metabolites identified in the isolated MEVs.

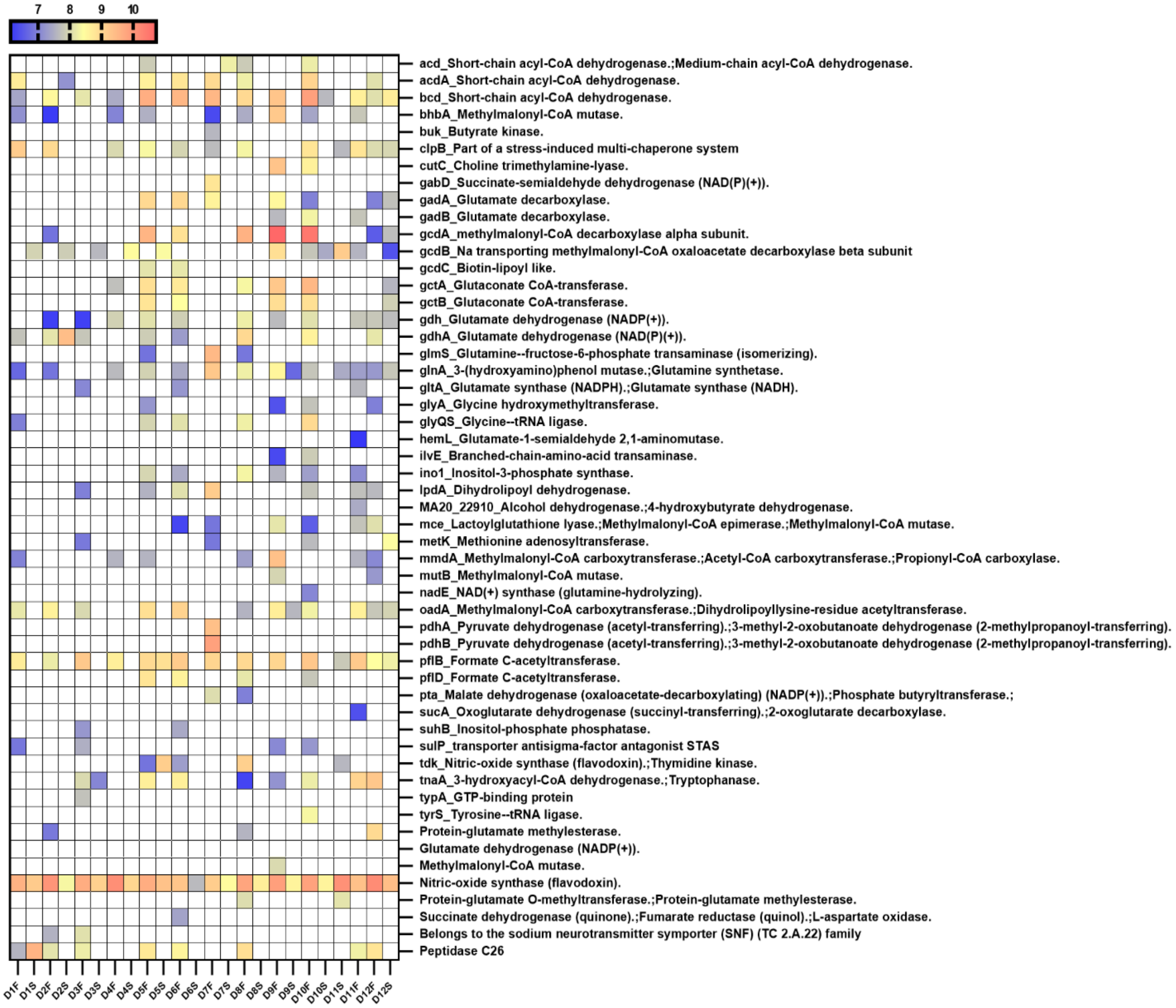

Figure S6. Identified Proteins in MEVs with neuroactive potential. The heatmap depicts the log10 of the summed protein intensities in the tested samples.

A

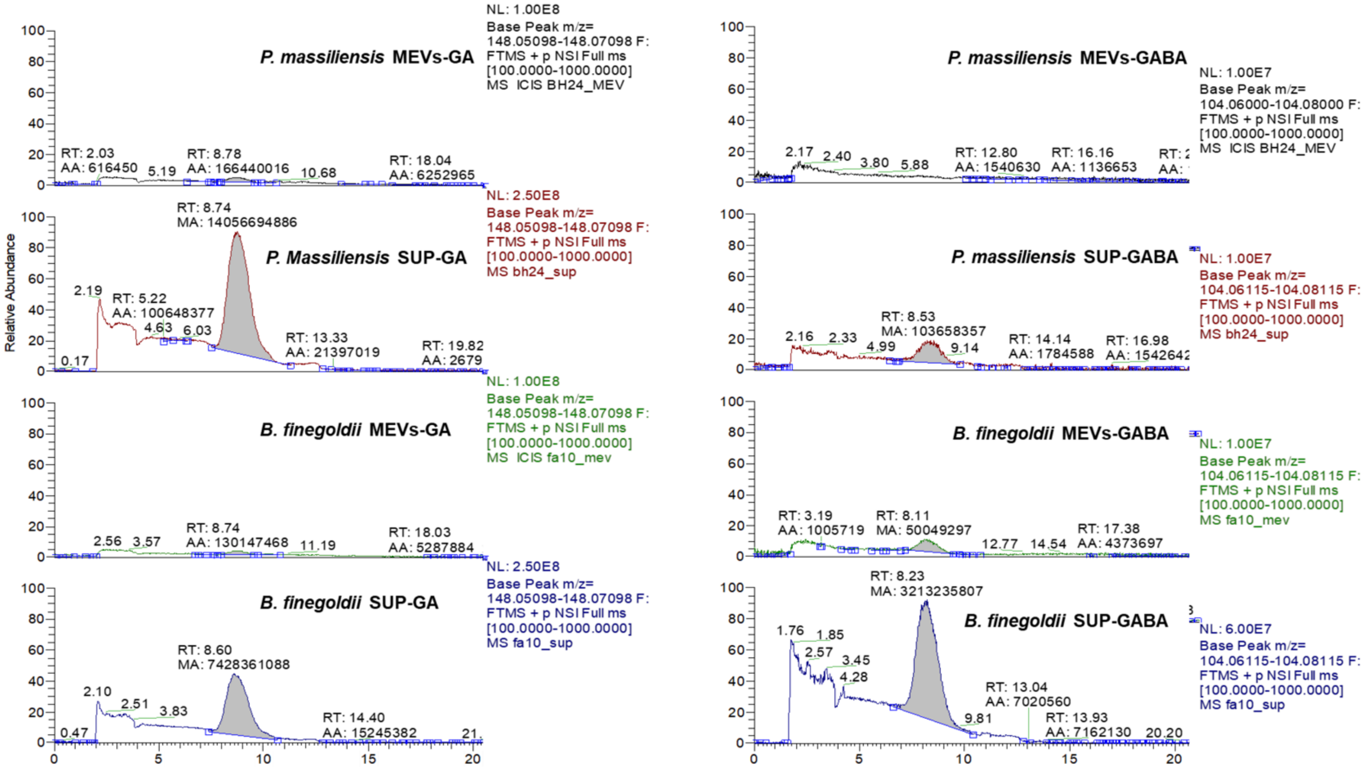

B

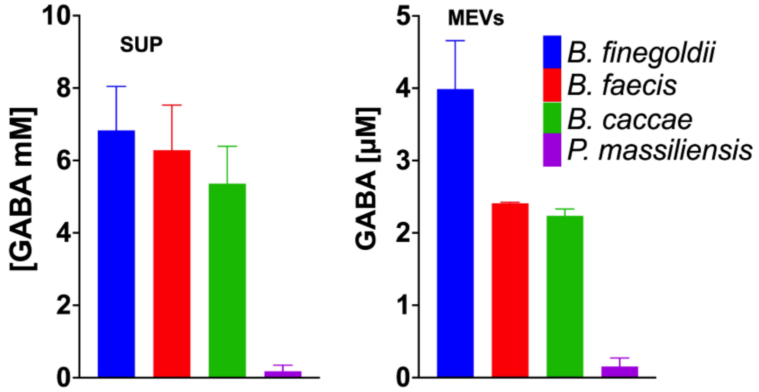

Figure S7. Comparative glutamic acid (GA) and GABA quantification based on extracted ion chromatography peak with a mass tolerance of 10 ppm in bacterial culture supernatants (**A**) vs generated EVs (**B**) from high and low GABA producing strains.

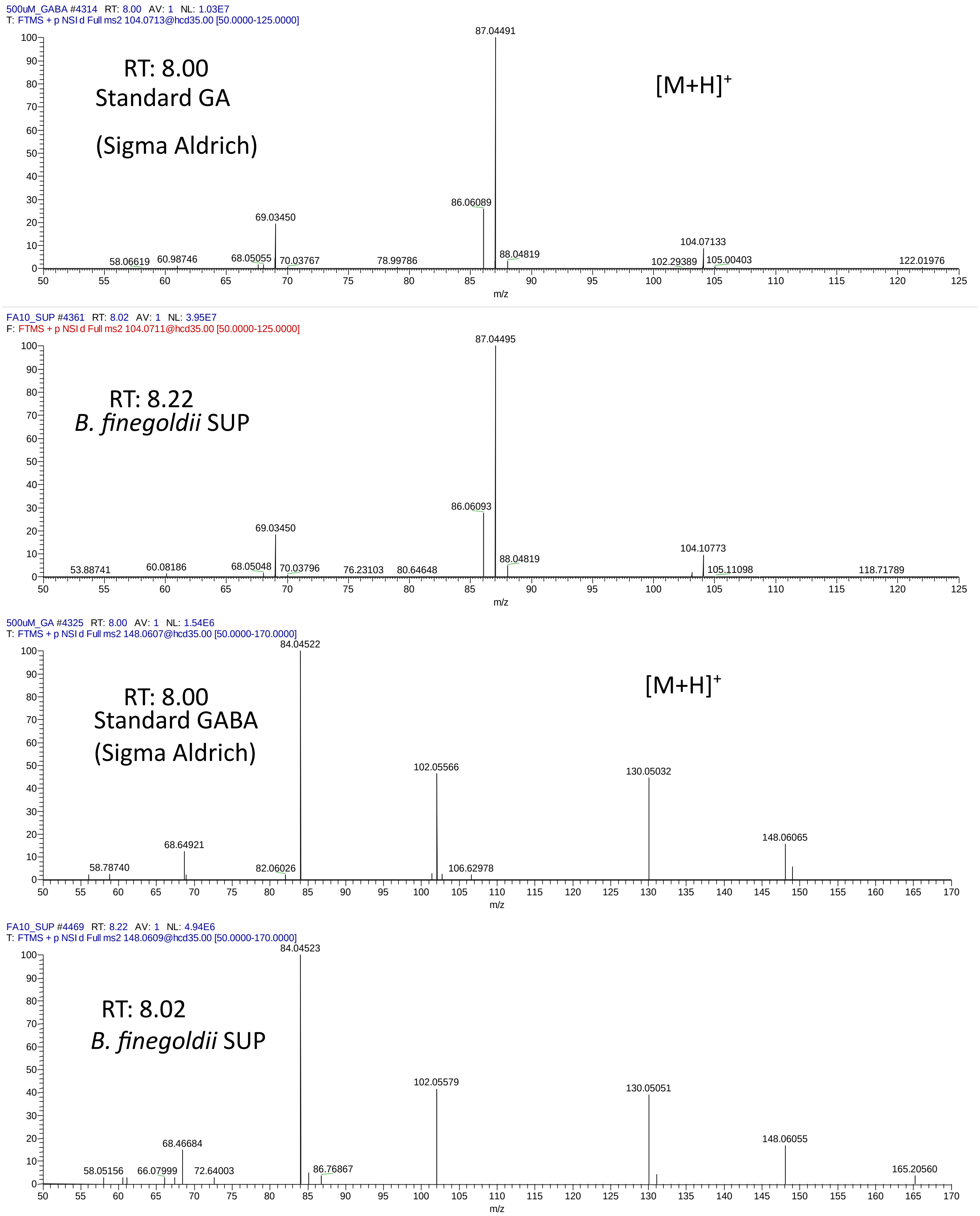

Figure S8. The fragmentation patterns of commercially available standards, GA and GABA, compared to identified corresponding compounds in *B. finegoldii* supernatant (SUP). Molecular ion is presented as $[M+H]^+$ and retention time as RT.

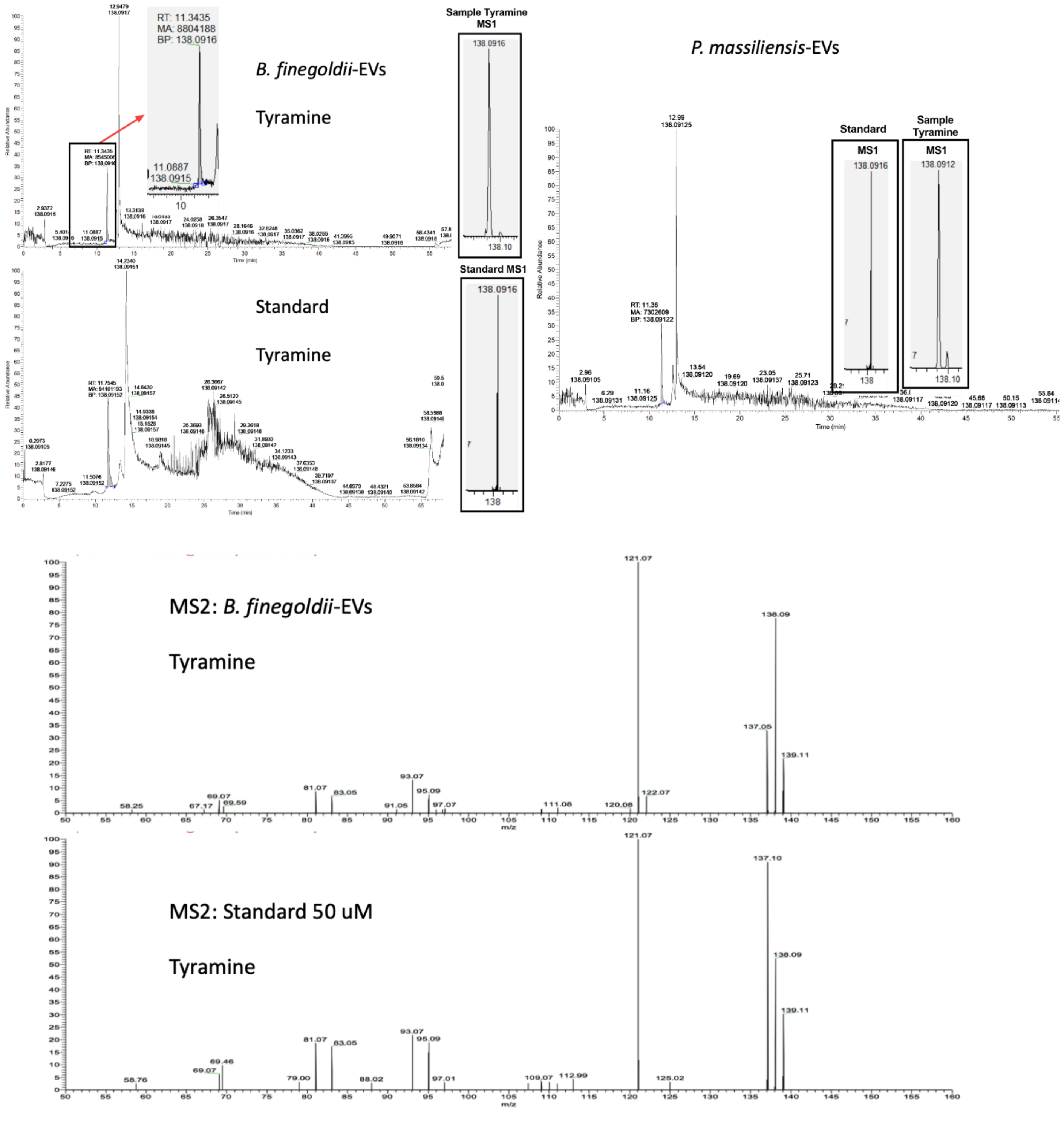

Figure S9. Quantification of tyramine based on extracted ion chromatography peak with a mass tolerance of 5 ppm in EVs extracted from *B. finegoldii* and *P. massiliensis.* (A) Chromatographic peaks of tyramine in *B. finegoldii* and *P. massiliensis* and standard. (B) Fragmentation patterns of standard and tyramine from *B. finegoldii* EVs.

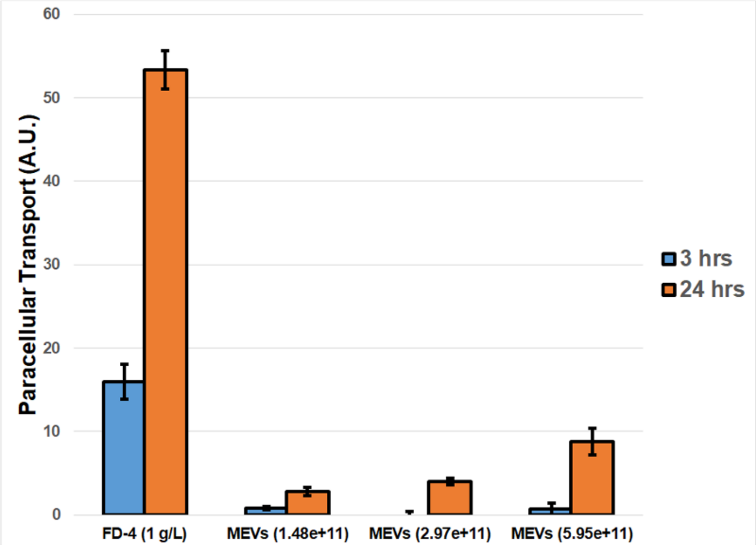

Figure S10. Paracellular transport of FITC-labeled MEVs as compared to FITC-dextran positive control across Caco-2 cell line after 3h and 24h of exposure.

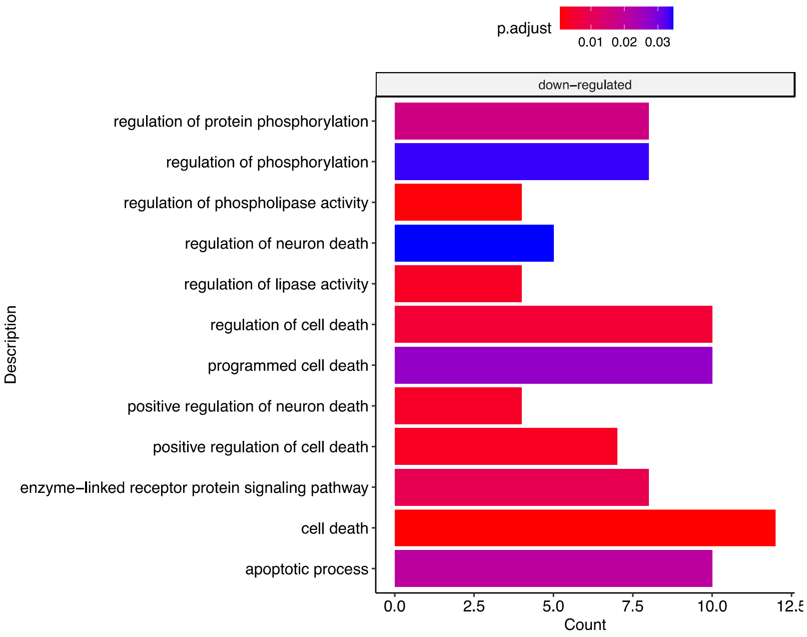

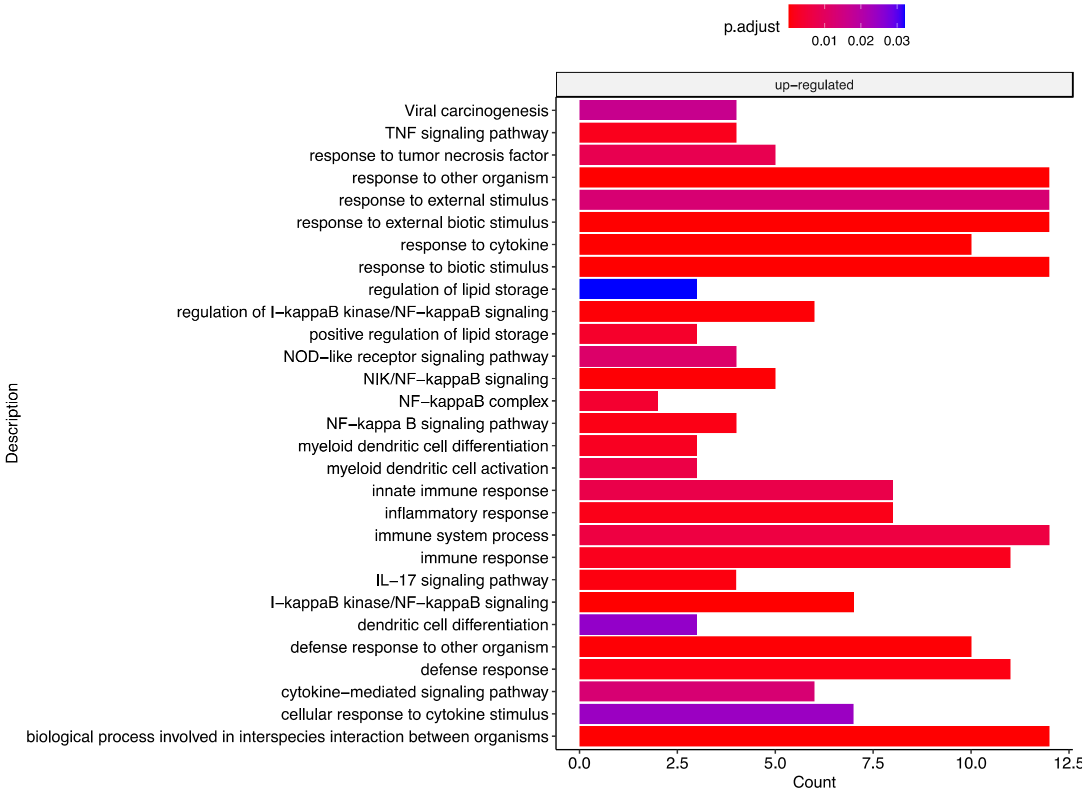

Figure S11. RNA-seq Functional Enrichment Analysis. Bar Plot illustrating functional enrichment of upregulated and downregulated genes in Caco-2 Cells following 24-hour exposure to MEVs with a p.adjusted threshold of 0.05.

A

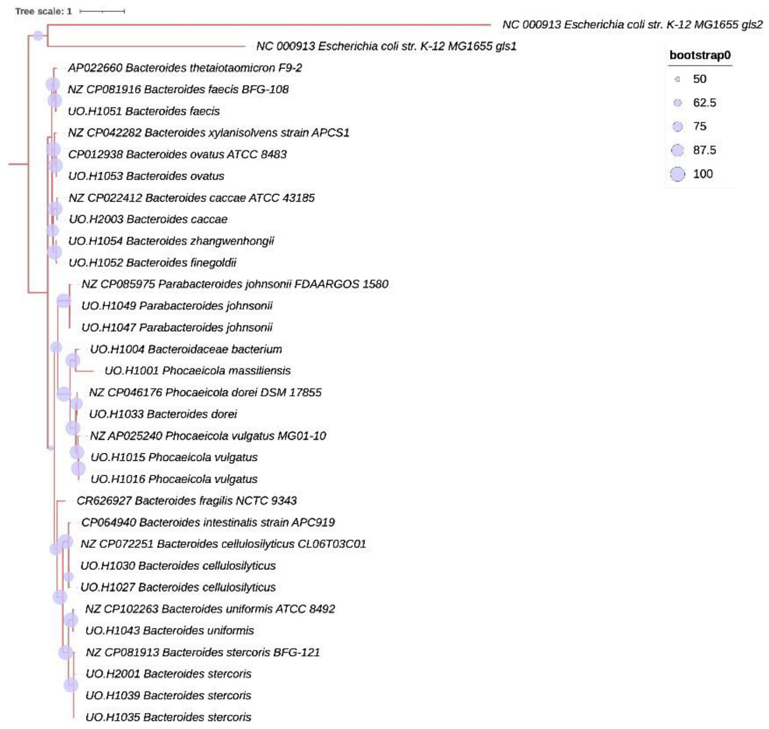

B

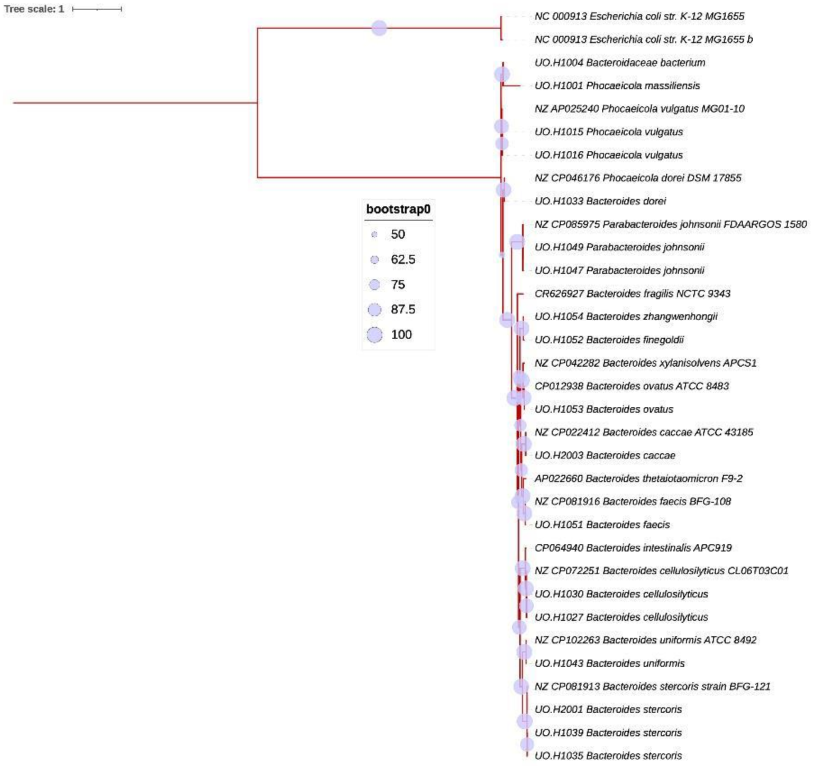

Figure S12: Phylogenetic clustering of the *Bacteroides* spp. isolates based on *gadA* (A) and *gadB* gene (B) sequences. The trees were constructed using IQ-TREE release 2.2.2.6[79] (http://www.iqtree.org/) and viewed using interactive Tree Of Life version 6 (iTOL V6, https://itol.embl.de/). Bootstrapping was performed with 1000 replicates, and the values are displayed as circles alongside the branches, representing the proportion of trees in which associated taxa formed distinct clusters.
